# Metagenomic analysis after selective culture enrichment of hospital and community wastewater enhances antimicrobial resistance gene detection

**DOI:** 10.1128/mbio.01672-25

**Published:** 2025-07-31

**Authors:** Nicole Acosta, Jangwoo Lee, Maria A. Bautista, Srijak Bhatnagar, Carmen Li, Barbara J. Waddell, Emily Au, Puja Pradhan, Rhonda G. Clark, Jon Meddings, Gopal Achari, Johann D. Pitout, John Conly, Kevin Frankowski, Casey R. J. Hubert, Michael D. Parkins

**Affiliations:** 1 Department of Microbiology, Immunology and Infectious Diseases, University of Calgary 198998, Calgary, Canada; 2 Department of Biological Sciences, University of Calgary 98634, Calgary, Canada; 3 Faculty of Science and Technology, Athabasca University 662869, Athabasca, Alberta, Canada; 4 Department of Medicine, University of Calgary and Alberta Health Services 2129, Calgary, Canada; 5 Department of Civil Engineering, University of Calgary 468050, Calgary, Canada; 6 Department of Pathology and Laboratory Medicine, University of Calgary and Alberta Health Services 574842, Calgary, Canada; 7 O'Brien Institute for Public Health, University of Calgary 157746, Calgary, Canada; 8 Infection Prevention and Control, Alberta Health Services 3146, Calgary, Canada; 9 Snyder Institute for Chronic Diseases, University of Calgary and Alberta Health Services 157743 , Calgary, Canada; 10 Advancing Canadian Water Assets, University of Calgary 2129, Calgary, Canada; Department of Biochemistry & Biomedical Sciences, McMaster University, Hamilton, Ontario, Canada

**Keywords:** antibiotic resistance, antimicrobial agents, metagenomic, wastewater

## Abstract

Antimicrobial resistance is an accelerating threat to global health. Wastewater-based surveillance (WBS) enables objective, inclusive, and comprehensive assessments of population-level antimicrobial resistance; however, it is limited in its ability to detect rare antibiotic resistance genes (ARG). We compared traditional high-depth metagenomic sequencing of raw wastewater with lower-depth sequencing following semi-selective culture enrichment for gram negatives for rare ARGs in wastewater from two tertiary-care hospitals and two nearby urban neighborhoods. In total, 26 antibiotic resistance gene types (1,225 subtypes) were identified, with beta-lactamase genes being the most prevalent. Resistomes differed between raw and culture-enriched wastewater metagenomes and clustered based on sample type (hospitals versus neighborhoods). Hospital wastewater had higher diversity and a greater abundance of ARGs relative to both raw and culture-enriched neighborhood wastewater metagenomes. Lower coverage sequencing following culture enrichment proved superior to deeper sequencing for identifying rare, clinically relevant targets, including carbapenemase genes. In particular, enrichment with meropenem proved the most sensitive to identifying clinically relevant genes and enabled significant cost savings. ARG WBS has enormous potential for augmenting hospital-based infection prevention and control and antimicrobial stewardship programs.

**IMPORTANCE:**

Antimicrobial resistance (AMR) poses a considerable burden to healthcare systems and contributes to increased morbidity and mortality. This is expected to further increase with time. AMR surveillance programs are key to understanding and controlling this progressive threat. Wastewater-based surveillance (WBS) is an emerging tool that can be adapted to this end. This study explores the role of metagenomic analysis of WBS with/and without culture enrichment to detect rare antibiotic resistance genes (ARG) of clinically important pathogens across a range of scales. We were able to demonstrate that the resistome of hospitals significantly differs from communities having a greater abundance, and more heterogeneous ARGs. Culture enrichment, particularly with meropenem, improved the detection of clinically relevant ARGs even at lower sequencing depths. WBS is an important tool with the capacity to augment hospital-based infection control and antimicrobial stewardship programs, providing real-time, cost-effective information on the population within.

## INTRODUCTION

The emergence and spread of antimicrobial-resistant organisms (ARO) is a profound global health threat (1). ARO lead to disproportionally higher morbidity and mortality, manifesting in huge healthcare costs (2). Globally, deaths related to ARO are expected to rise to 10 million per year by 2050, costing the global economy >$100 trillion in lost productivity annually (3). Emerging threats include Enterobacterales and *Pseudomonas aeruginosa,* which produce extended-spectrum beta-lactamases (ESBLs) (4–7), metallo-beta-lactamases (MBLs) (8–13), and *Klebsiella pneumoniae* carbapenemases (KPC) (12, 14, 15). These infections are increasing in incidence and are confounded by limited (occasionally none) treatment options. Accordingly, these pathogens are recognized as key organisms of concern by the World Health Organization and the US Centers for Disease Control and Prevention (16–18).

Recently, Mitchell and collaborators (19) performed an exploratory analysis of antimicrobial resistance (AMR) and proposed a framework to align AMR research with policy and its connections across the One Health landscape (i.e., humans–animals–environment). Two of the eight key themes emphasized were the optimization of antimicrobial resistance surveillance and understanding it in the environment (19). These key aspects require a broad knowledge of resistance burden within human populations and understanding of factors that contribute to AMR outbreaks and hotspots. Taking this into consideration, wastewater-based surveillance (WBS) is a unique tool that can be adapted for this purpose. Indeed, WBS has proven to be a transformative technology for understanding and modeling the COVID-19 pandemic (20–26). Advantages of WBS include its ability to serially monitor entire populations passively and comprehensively, providing objective, inclusive, and cost-effective public health data (27, 28).

Multiple approaches have been used in initial investigations of antibiotic resistance genes (ARGs) in wastewater, including culture-dependent or -independent approaches. These works have identified clinically relevant ARGs in wastewater, including metallo-beta-lactamases (MBLs) (e.g., *bla*_NDM_, *bla*_VIM_, *bla*_IMP_) and mobile colistin resistance (e.g., *mcr-1*) (29, 30). A limitation of metagenomic assessment of environmental samples, such as wastewater, relates to the sequencing depth required to increase sensitivity for low-abundance targets. This can be, to a limited degree, mitigated with greater sequencing depth but manifests in much higher costs to generate these larger libraries (31, 32). This is further complicated by the high prevalence of molecular inhibitors and non-target DNA from other organisms in wastewater. In order to overcome some of these limitations and improve the detection of ARGs, limited selective culture enrichment prior to sequencing has been incorporated into metagenomic assessments of different samples (e.g., sputum, oceanic sediments, drinking water, wastewater influent and effluent samples, and treated wastewater-irrigated soils) (33–37). However, studies assessing wastewater from hospitals that use differential enrichment to identify clinically relevant antibiotic resistance have not been performed. Manipulating growth conditions can ensure specific phenotypes of interest (i.e., those mediated via carbapenemases) to be enriched relative to non-target DNA, thereby improving detection limits of agnostic metagenomics, potentially facilitating the detection of low-abundance genes representing low-burden colonization impacting a small number of individuals while producing cost savings (38, 39). Developing and applying these strategies in hospitals is particularly important, as hospitals are where those most likely to acquire and experience adverse outcomes from antibiotic-resistant organisms exist (40, 41) and therefore represent the population most likely to derive benefit from new technologies to understand and mitigate AMR spread.

This study compared the resistomes generated from raw, deep-sequenced wastewater from two tertiary care hospitals and two nearby neighborhoods in Calgary, Canada with lower-depth sequencing after semi-selective enrichment using four different treatment conditions in order to identify a strategy amenable to future long-term surveillance efforts. New tools that serially and comprehensively assess AMR across entire hospital populations to augment infection prevention and control and antimicrobial stewardship programs are required, particularly those that target the silent reservoir of highly important ARGs. The aims of this work were to establish if the resistome of hospital wastewater differs from the general population, demonstrate that selective culture enrichment increases the potential for metagenomic sequencing to detect rare ARG of clinically relevant gram-negative pathogens, and determine the most effective enrichment condition.

## RESULTS

### General composition profiles of ARGs in wastewater samples

**Fig 1:**
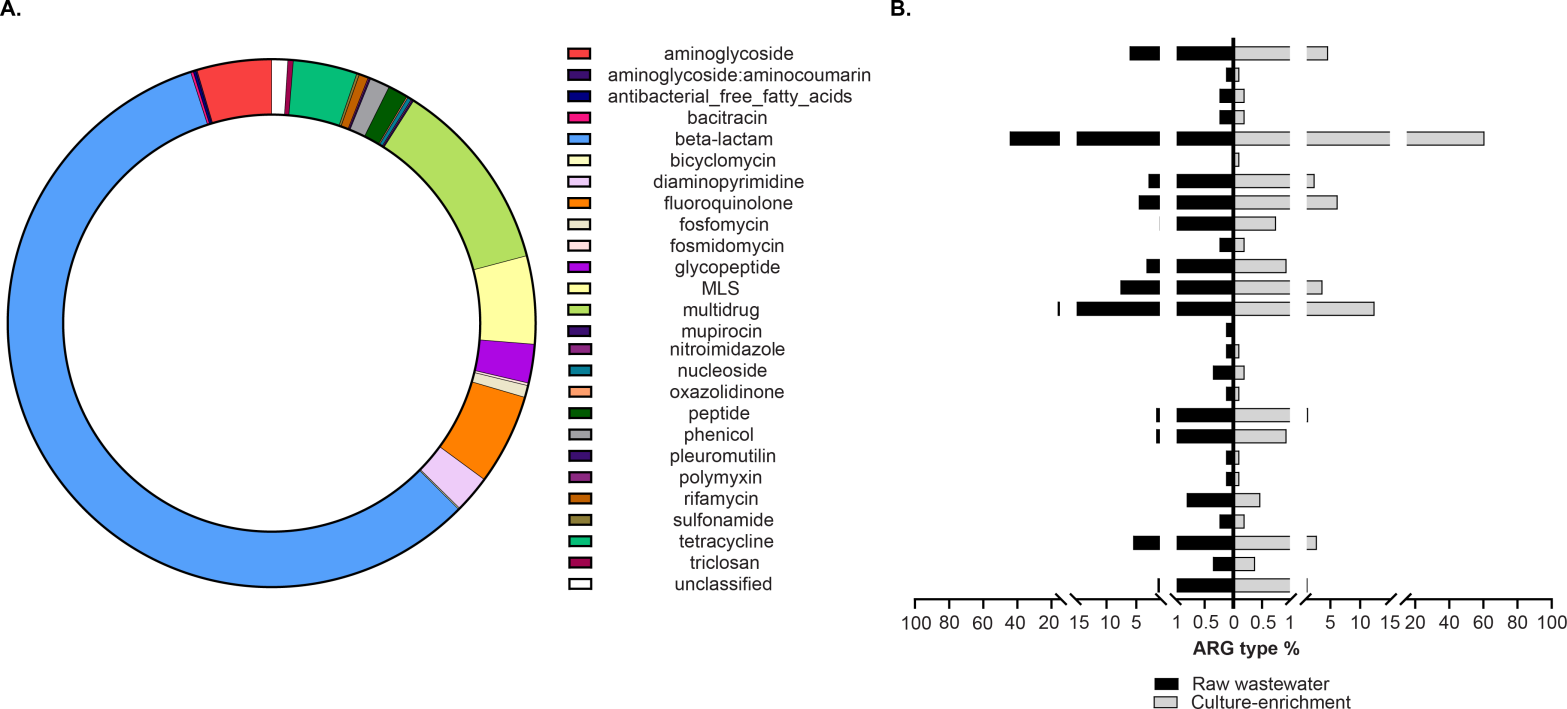
Composition profile of ARG types in wastewater. (**A**) Percentage of the resistome for each ARG type (mechanisms for antibiotic resistance) found in all wastewater samples (before and after culture enrichment) from both hospital and neighborhood sampling locations. (**B**) ARG type percentage classified by the type of sample (i.e., raw wastewater and culture enrichment).

ARG profiles were assessed in metagenomic libraries constructed before and after selective culture enrichment of gram-negative pathogens present in raw wastewater (hospital and neighborhood, Fig. S1) by inoculating them onto MacConkey agar augmented with sub-inhibitory concentrations of meropenem, ciprofloxacin, ceftriaxone, or colistin. Shotgun sequencing yielded 1.24 billion raw reads; 31.8% and 68.2% of those sequences were obtained from raw wastewater (*n* = 4) and selective culture-enrichment samples (*n* = 16), respectively. Mean library sizes were 98.6 ± 22.1 million raw reads per raw wastewater sample, and 49.7 ± 13.7 million reads per culture-enriched sample (Table S1). Out of all sequenced reads, 5.3 million raw reads (0.4%) were successfully aligned to the DeepARG database (42). Out of the 30 ARG types described in the DeepARG database (42), 26 comprising 1,225 ARG subtypes (Dataset S1) were identified. The ARG types encompassing the greatest diversity of subtypes were beta-lactam resistance (57.6%), followed by multidrug (11.8%), fluoroquinolone (5.6%), macrolide-lincosamide-streptogramin (MLS) (5.4%), aminoglycoside (4.6%), tetracycline (3.9%), and glycopeptide (2.4%) resistance (Fig. 1A).

### Composition profiles of ARGs in wastewater differ before and after selective culture enrichment

Differences in the proportions and distributions of ARG types in raw wastewater and culture-enriched samples were observed (Fig. 1B). The proportion of beta-lactam resistance gene types identified following selective enrichment was significantly greater than that in raw wastewater sequenced directly (adjusted *P*-value < 0.05). In contrast, multidrug, MLS, tetracycline, and glycopeptide ARG types, less common in gram negatives, were more prevalent in raw wastewater (adjusted *P*-value < 0.05). Although the median Shannon diversity index for hospitals (i.e., 4.9) samples was higher than that of the neighborhood (i.e., 4.5) samples, it was not statistically significant (*P* = 0.333, Mann-Whitney test). To assess selective culture enrichment with lower sequencing depth as a suitable approach for identifying and improving ARG detection in wastewater, normalized gene abundance ratios were calculated for ARG types after selective culture enrichment to determine an Enrichment Factor (EF), the ratio by which ARGs increased or decreased relative to raw wastewater. Out of the 26 ARG types found in this study (Fig. 1), 16 increased in relative abundance compared with raw wastewater regardless of the antibiotic that was used for selective enrichment (Table 1). ARG types experiencing the greatest increase after differential culture enrichment were polymyxin (median EF 23.9, interquartile range [IQR] 12.1–37.9), followed by aminoglycoside:aminocoumarin and nitroimidazole. Additionally, bicyclomycin was the only ARG type that was exclusively detected in culture-enriched wastewater. In contrast, tetracycline, bacitracin, and rifamycin types were least enriched. Several ARG types associated with gram positives were found to have EF decrease after culture enrichment, regardless of the conditions tested (i.e., glycopeptide) or in neighborhood samples for all enrichment conditions except with colistin (i.e., antibacterial free fatty acids and pleuromutilin) (Table 1). Similarly, some gram-positive ARG types were identified exclusively in raw wastewater metagenomes and not following enrichment on MacConkey agar. Of those, mupirocin was the only ARG type that was observed for all conditions and locations tested (Table 1). However, its normalized abundance was among the lowest observed in raw wastewater (i.e., 7.1 × 10^−5^). Similarly, ARGs for oxazolidinones were only found in raw wastewater metagenomes, with the exception of two samples (Table 1). Pleuromutilin and antibacterial free fatty acid ARG types were similarly detected only in raw wastewater from hospitals (i.e., Hospitals 1 and 2) (Table 1).

**TABLE 1:** Changes in ARG types after culture enrichment of wastewater on MacConkey agar plates supplemented with selective antibiotics relative to pelleted wastewater^*e*^

	Enrichment factor (EF)^*a*^															
	Hospitals								Neighborhoods							
	Hospital-1				Hospital-2				NE Neighborhood				NW Neighborhood			
ARG types (n)^*b*^	MEM	CIP	CRO	COL	MEM	CIP	CRO	COL	MEM	CIP	CRO	COL	MEM	CIP	CRO	COL
Aminoglycoside (56)	1.5	2.0	1.8	2.9	2.8	2.5	3.2	3.7	6.8	8.8	11.0	3.8	0.9	6.4	7.1	3.6
Aminoglycoside-aminocoumarin (1)	35.8	30.1	22.6	27.3	18.2	10.1	12.4	11.2	14.4	27.8	35.6	23.2	0.0^*d*^	38.5	33.7	42.1
Antibacterial free fatty acids (2)	0.0	0.0	0.0	0.0	0.0	0.0	0.0	0.0	0.0^*d*^	0.0^*d*^	0.0^*d*^	0.0	0.1	0.1	0.1	0.0
Bacitracin (2)	1.4	1.4	1.2	1.3	1.8	1.5	1.5	1.6	3.2	3.0	3.3	1.5	1.7	2.4	2.6	1.4
Beta-lactam (705)	1.5	1.8	1.7	1.7	3.5	2.6	2.6	1.7	2.3	3.7	5.5	1.5	0.1	3.8	4.8	1.6
Bicyclomycin (1)	**FNQ**	0.0	**FNQ**	0.0	0.0	0.0	**FNQ**	0.0	**FNQ**	0.0	0.0	**FNQ**	**FNQ**	0.0	**FNQ**	**FNQ**
Diaminopyrimidine (28)	5.3	6.5	7.8	2.3	9.4	6.3	9.8	0.2	19.8	7.9	9.6	2.3	3.2	15.8	18.7	3.2
Fluoroquinolone (68)	5.2	4.5	4.5	3.2	5.2	3.4	4.2	1.6	8.1	17.8	21.2	3.8	0.2	22.9	19.3	6.5
Fosfomycin (9)	6.3	8.4	4.8	11.8	1.6	7.8	9.6	0.3	0.4	6.8	5.4	0.7	0.2	13.2	4.0	3.7
Fosmidomycin (2)	12.9	11.6	10.0	11.8	7.0	5.0	5.6	3.6	7.4	13.1	16.4	13.0	0.8	14.3	12.6	17.0
Glycopeptide (29)	0.2	0.1	0.1	0.0	0.3	0.0^*d*^	0.1	0.0^*d*^	3.8	0.0^*d*^	0.0^*d*^	0.0	0.3	0.1	0.3	0.0^*d*^
MLS (66)	1.0	0.8	0.8	0.4	1.5	1.3	1.9	1.1	0.7	0.8	1.1	0.5	0.1	0.5	0.6	0.4
Multidrug^*c*^ (145)	6.4	5.8	4.9	4.3	5.0	3.0	3.6	2.0	6.5	6.2	8.7	3.2	2.0	3.9	5.1	2.5
Mupirocin (1)	0.0	0.0	0.0	0.0	0.0	0.0	0.0	0.0	0.0	0.0	0.0	0.0	0.0	0.0	0.0	0.0
Nitroimidazole (1)	24.7	22.6	20.2	24.5	9.4	6.0	7.1	7.4	9.0	13.8	18.1	15.1	1.5	21.4	21.0	25.7
Nucleoside (3)	0.1	2.3	2.2	0.7	1.8	3.7	8.2	0.4	3.2	5.1	5.2	20.5	1.1	6.1	39.0	10.1
Oxazolidinone (1)	0.0	0.0	0.0	0.0	0.0	0.0	0.0	0.0	0.9	0.0	0.0	0.0	6.6	0.0	0.0	0.0
Peptide (15)	9.5	9.4	8.8	6.5	7.9	4.7	5.5	1.1	6.3	10.4	14.2	3.0	0.7	9.4	9.8	3.3
Phenicol (15)	0.5	1.3	1.6	9.4	2.2	3.2	4.7	6.2	0.9	5.4	4.9	7.9	0.2	3.8	6.2	7.8
Pleuromutilin (1)	0.0	0.0	0.0	0.0	0.0	0.0	0.0	0.0	0.0	0.0	0.0	0.0	0.1	0.0^*d*^	0.0^*d*^	0.0
Polymyxin (1)	50.2	49.3	37.9	39.0	16.0	10.7	12.1	11.3	11.3	22.4	23.9	22.8	4.9	29.5	31.0	36.3
Rifamycin (7)	1.0	2.4	3.6	0.1	1.3	4.2	3.7	0.0^*d*^	0.1	0.8	0.4	0.2	0.1	0.3	0.7	0.0^*d*^
Sulfonamide (2)	1.8	2.8	2.0	2.5	2.6	2.2	2.8	1.6	12.8	7.8	9.8	1.3	1.2	7.4	14.3	1.3
Tetracycline (48)	1.1	1.0	1.0	1.5	1.1	1.4	1.7	4.6	3.5	2.5	3.6	1.3	0.2	2.4	2.8	1.5
Triclosan (4)	25.6	0.3	0.6	0.2	0.4	0.2	0.3	0.0^*d*^	310.7	0.2	1.5	0.3	58.9	0.3	26.7	0.2
Unclassified (12)	8.4	7.7	7.7	7.5	8.9	5.6	6.6	5.0	9.9	16.2	21.3	10.6	1.0	20.6	19.6	16.3

^*a*^ Enrichment factor (EF) was calculated as the ratio of a given ARG type (normalized by total 16S rRNA) after culture enrichment relative to raw wastewater. EF >1 indicates the abundance of the ARG increased, whereas EF <1 (in gray) indicates ARG abundance decrease. EF = 0 indicates that a given ARG type was only found in the raw wastewater. ARG types in enriched samples with no corresponding reads to compare against from raw wastewater are indicated by non-quantifiable (FNQ) (bold) (i.e., cannot divide by zero).

^*b*^ n denotes the number of ARG subtypes detected for each ARG type.

^*c*^ Multidrug ARG type contains genes that confer resistance to multiple antibiotic categories, including macrolides, beta-lactamases, glycopeptides, quinolones, as well as other antimicrobials such as metals.

^*d*^ EF is less than <0.05.

^*e*^ Meropenem (MEM), ciprofloxacin (CIP), colistin (COL), and ceftriaxone (CRO).

**Fig 2:**
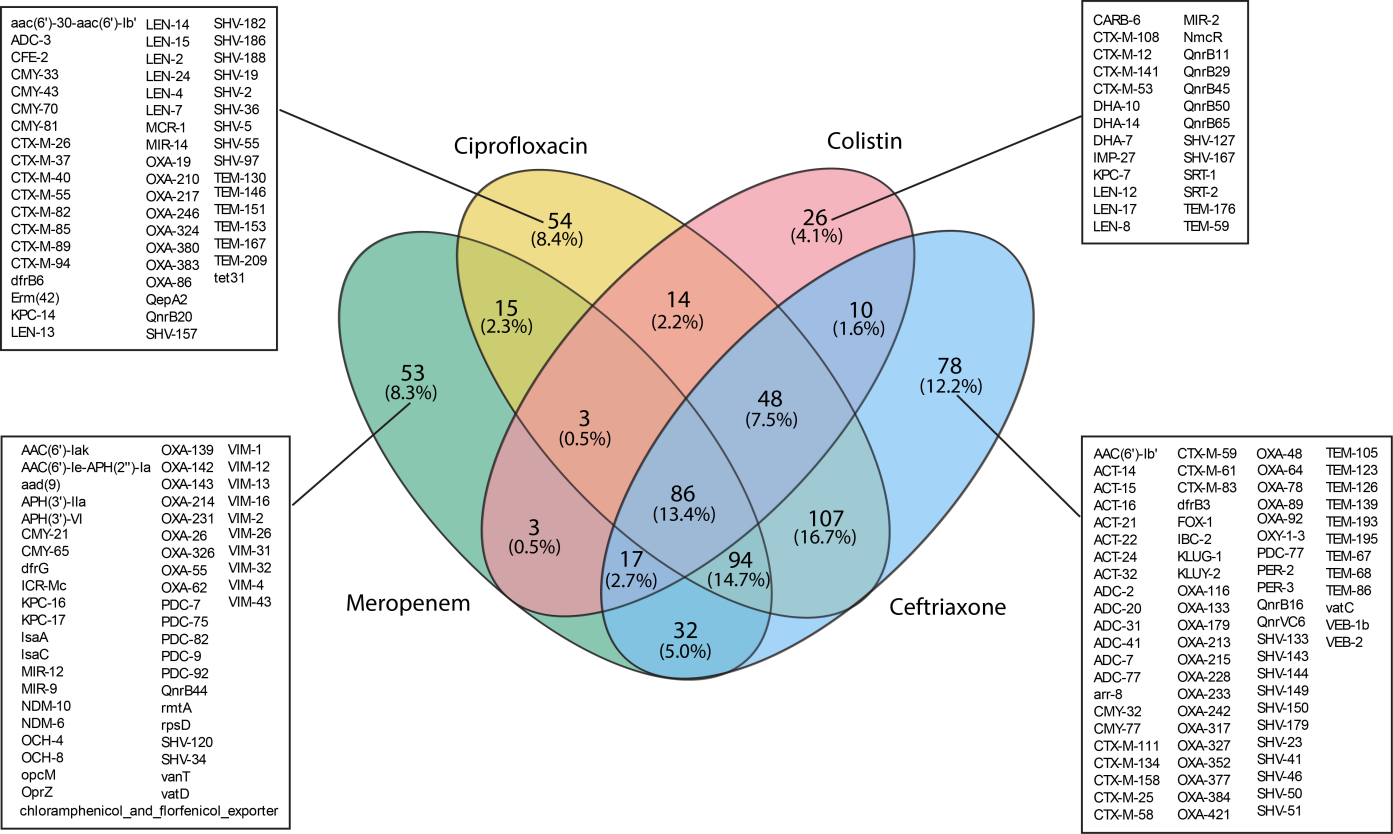
Cultured-enriched ARGs classified by the antibiotic used for selection. Common and unique ARG subtypes among the 640 genes that were only detected through culture-enrichment at one or more sites, where the ARGs were undetected in the corresponding raw wastewater and only detected after at least one enrichment condition. ARGs are classified by the antibiotic used for culture enrichment. Only those ARGs unique to a specific antibiotic enrichment condition are listed in individual boxes. Similar details about the ARGs shared between all four enrichment conditions (*n* = 86) are detailed in Fig. S3. ARGs that were found in two or three enrichment conditions are listed in Data set S3.

To further understand ARGs that are enriched after limited bacterial growth on antibiotic-impregnated MacConkey agar, we assessed data at the ARG subtype level. Among the 1,225 identified ARG subtypes (Data set S1), 640 were only detected through culture-enrichment at one or more sites, where the ARGs were undetected in the raw wastewater sample and only detected after at least one enrichment condition (termed as cultured-enriched ARGs, hereafter) (Data set S2). Cultured-enriched ARGs were classified into 16 different ARG types, the majority being beta-lactamase genes (80%) followed by fluoroquinolone (7%) and multidrug resistance genes (3%) (Fig. S2). Some enrichment conditions resulted in larger improvements in ARG detection compared with others; notably, ceftriaxone-impregnated media exhibited the highest effectiveness, uncovering 472 out of the 640 culture-enriched ARGs (*P* < 0.001). Furthermore, among ARGs uniquely identified by a single antibiotic enrichment condition, ceftriaxone gave rise to the most (78), whereas colistin yielded the least (26) (Fig. 2). Eighty-six ARGs were shared between the meropenem-, ciprofloxacin-, colistin-, and ceftriaxone-selective enrichment conditions (Fig. 2; Fig. S3), with 63% of these being beta-lactamase genes and predominantly represented by ESBLs including *bla*_TEM_ and *bla*_CTX-M_, as well as ARGs *bla*_CMY_, *bla*_ACT_, and *bla*_DHA_ (Fig. S3). Because one of the aims of the study was to identify rare, clinically relevant carbapenemases, cultured-enriched ARGs were classified as beta-lactamase or non-beta-lactamase genes (Fig. S4). Of the 640 culture-enriched ARGs, 511 were beta-lactamase genes, including 45 carbapenemases. Meropenem enrichment yielded the highest rate of carbapenemase detection, accounting for 88.9% of all culture-enriched carbapenemases, whereas colistin enrichment yielded the lowest (8.9%) (Fig. S4, Venn diagram). Similarly, among the remaining 129 non-beta-lactamase ARGs, meropenem proved to be the most sensitive in identifying other ARG types (Fig. S4, table). Therefore, we established a workflow that illustrates the overall effectiveness of enrichment conditions in uncovering clinically relevant resistance genes and identifying the most efficient condition for ARG recovery (Fig. S4).

To further analyze the differences based on sample type, the distribution of cultured-enriched ARGs was categorized by sample type independent of the antibiotic used for selection. Beta-lactamase genes, the most common cultured-enriched ARGs found, differed in proportion based on sample type (Hospitals 86.6% vs Neighborhoods 75.2%, *P* = 0.0048, Fisher exact tests). Fluoroquinolone ARGs were the next most prevalent, and their proportion was similar across location types (Hospitals 6.6% vs Neighborhoods 5.7%, *P* = 0.833, Fisher exact tests) (Fig. S2). Out of the total 640 genes that were identified in enriched samples but undetected in the paired raw wastewater from a given site, 376 ARGs were also not detected in any of the other raw wastewater sites, meaning that these ARGs could only be detected through culture enrichment (Fig. S5).

**Fig 3:**
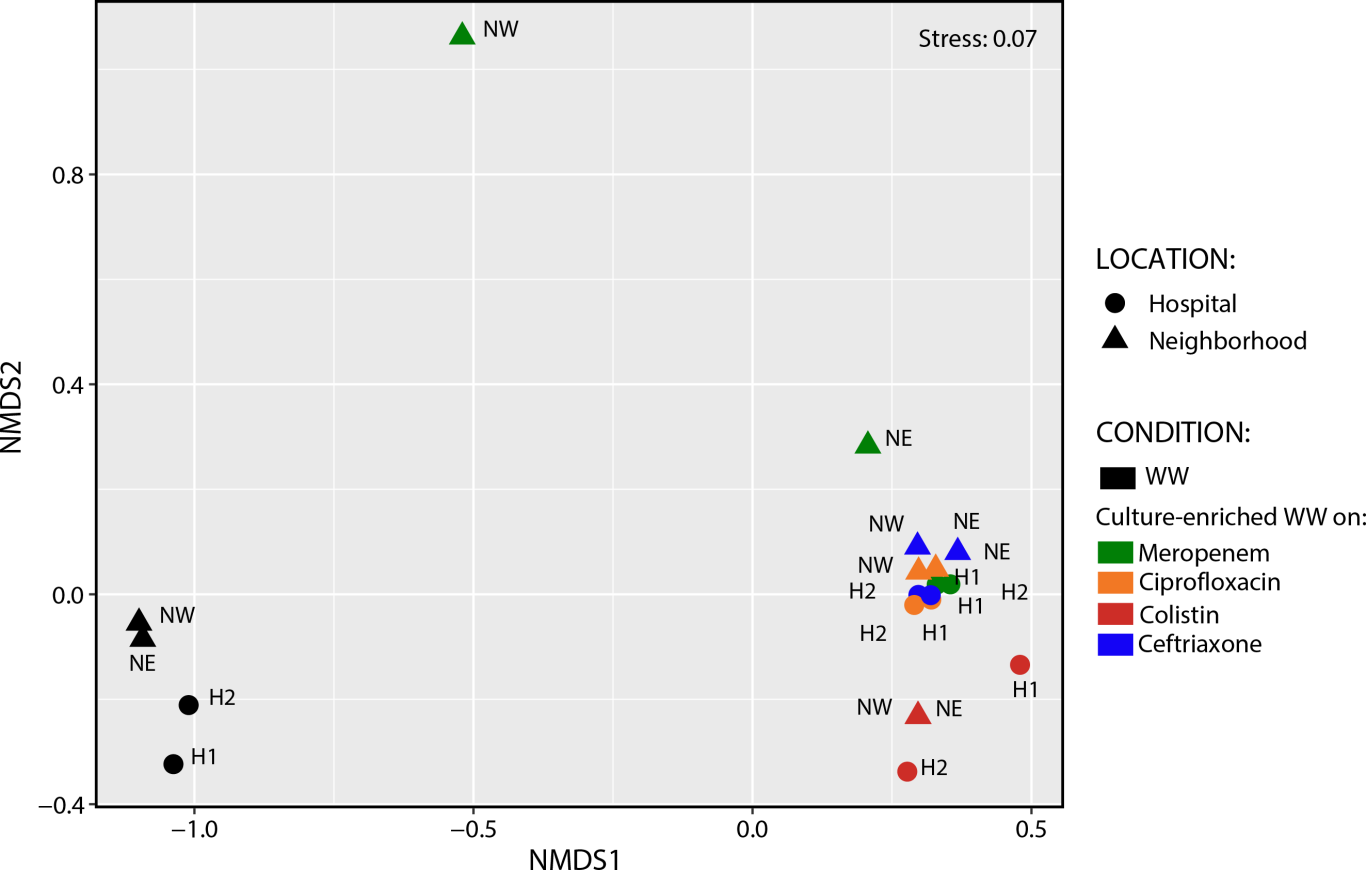
Non-metric multidimensional scaling (NMDS) of wastewater ARG beta-diversity before and after culture enrichment. NMDS was done on the Bray-Curtis distances of ARG profiles. Symbol shape denotes the sampling location [i.e., hospital (H1: Hospital-1 and H2: Hospital-2) or neighborhood (NE and NW)], and color denotes enrichment conditions (i.e., raw wastewater versus different selective media). WW: raw wastewater.

To better understand the differences in resistomes across locations and conditions, we calculated the Bray-Curtis dissimilarities and found a clustering of samples based on treatment (i.e., before and after culture enrichment) and sample location (i.e., hospital and neighborhood) (Fig. 3). We performed the ANOSIM test and found that overall beta diversity for different enrichment treatments (ANOSIM statistic R = 0.799, *P* = 0.0012) and sampling locations (ANOSIM statistic R = 0.112, *P* = 0.0463) were significantly different. To further test the effect of treatment and sample location, we performed a PERMANOVA to determine the effect of those variables on the resistome. ARG subtype profiles were found to be more distinguishable by treatment (R^2^ = 41.7, *P* = 0.001), whereas collection location did not pass statistical significance thresholds (R^2^ = 9.6, *P* = 0.09). Although most samples were tightly clustered by treatment, an outlier was found in the neighborhood samples plated on meropenem where the two neighborhoods deviated significantly (Fig. 3). To ascertain which ARGs in NW-meropenem differed from NE-meropenem, biplot analysis was performed revealing 16 ARGs that were significantly associated with one neighborhood or the other (*P* ≤ 0.001) (Fig. S6). Further analysis using a heatmap for the selected 16 ARGs revealed that six genes, including one aminoglycoside (*ant* (9)-I), one MLS (*erm*(TR)), three multidrug (*lsa*, *efrA*, and *efrB*), and one oxazolidinone (*optrA*), were specifically associated with NW-meropenem (Fig. S7), whereas other ARGs were disproportionally abundant but not unique to NW.

### Beta-lactamase diversity from hospital and community cultured-enriched samples

**Fig 4:**
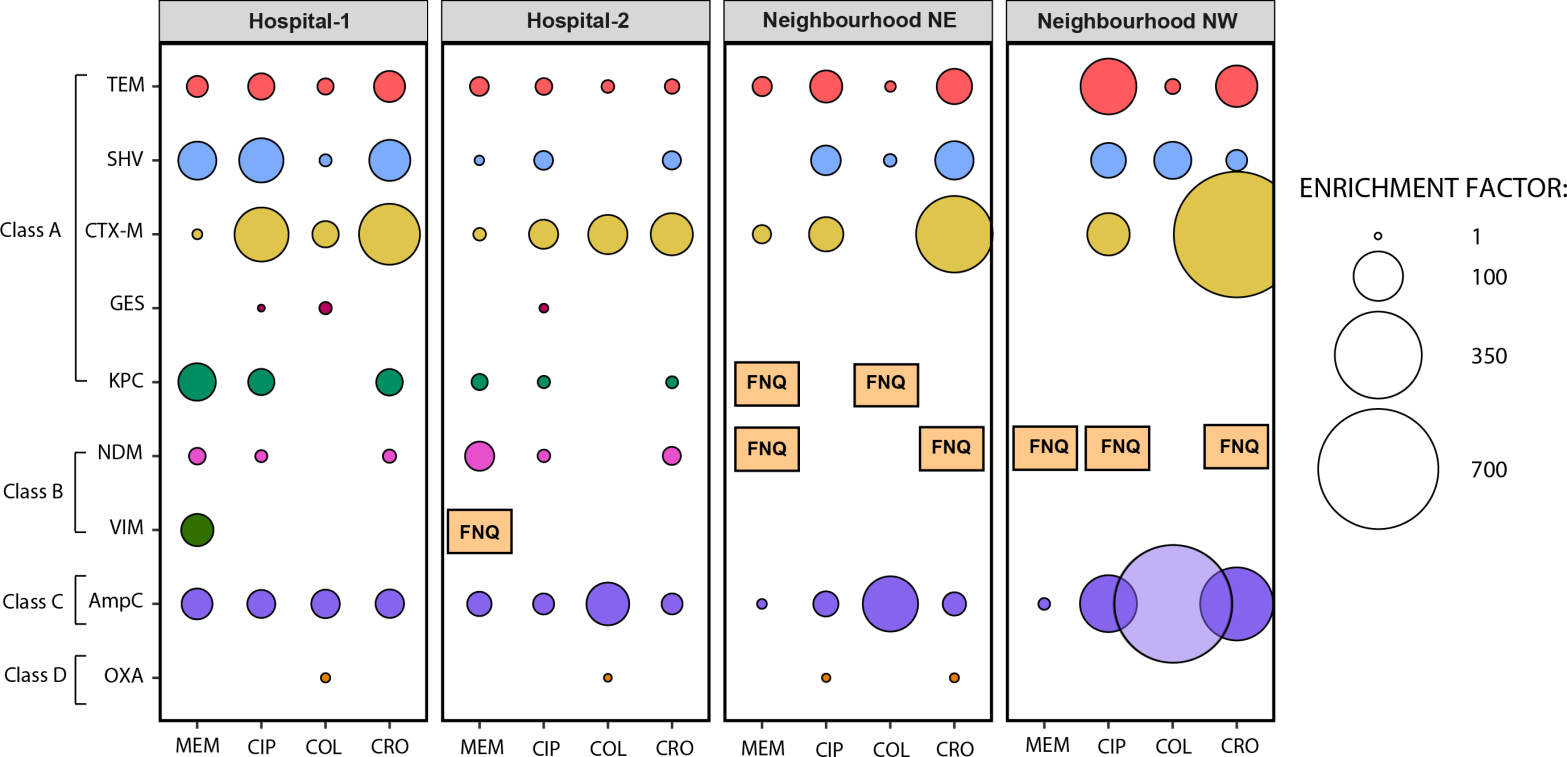
Relative enrichment of β-lactamases found in the wastewater resistome after selective culture enrichment. Enrichment factor (EF) (shown as bubbles) was calculated as the metagenomic abundance ratio of any given β-lactamase:16S rRNA genes following growth on MacConkey agar plates impregnated with antibiotics relative to raw wastewater samples. Circles are shown only when EF >1. Factor non-quantifiable (**FNQ**) represents ARGs only identified in enriched samples as raw wastewater samples contained 0 reads for the beta-lactamase ARG. MEM: meropenem, CIP: ciprofloxacin, COL: colistin, and CRO: ceftriaxone.

Because beta-lactam resistance was identified as the most diverse ARG type and given its clinical importance, we further analyzed the 16S rRNA gene-normalized abundance of specific beta-lactamase genes before and after culture enrichment. Beta-lactamases were grouped by the Ambler classification, and EFs were calculated. Culture enrichment was especially strategic in uncovering carbapenemases such as *bla*_KPC_, *bla*_NDM_, and *bla*_VIM_ that were not detected in corresponding metagenomes from raw wastewater, even when larger libraries were obtained for raw wastewater at certain locations (Fig. 4; Table S1). Hospitals yielded the greatest abundance and beta-lactamase diversity. Interestingly, *bla*_VIM_ was only detected in hospital samples selectively enriched after growth on meropenem-MacConkey. The average enrichment factors from each pairing for *bla*_KPC_ and *bla*_NDM_ from meropenem-impregnated MacConkey samples were 29.1 and 17.4, respectively (Fig. S8). *bla*_CTX-M_ and *bla*_AmpC_ had the highest average EF values regardless of the antibiotic used (101 and 90 EF, respectively). A few beta-lactamases (i.e., *bla*_OXA_ and *bla*_GES_) were found to have decreased in abundance after culture enrichment in some conditions (Fig. 4; Fig. S8).

**Fig 5:**
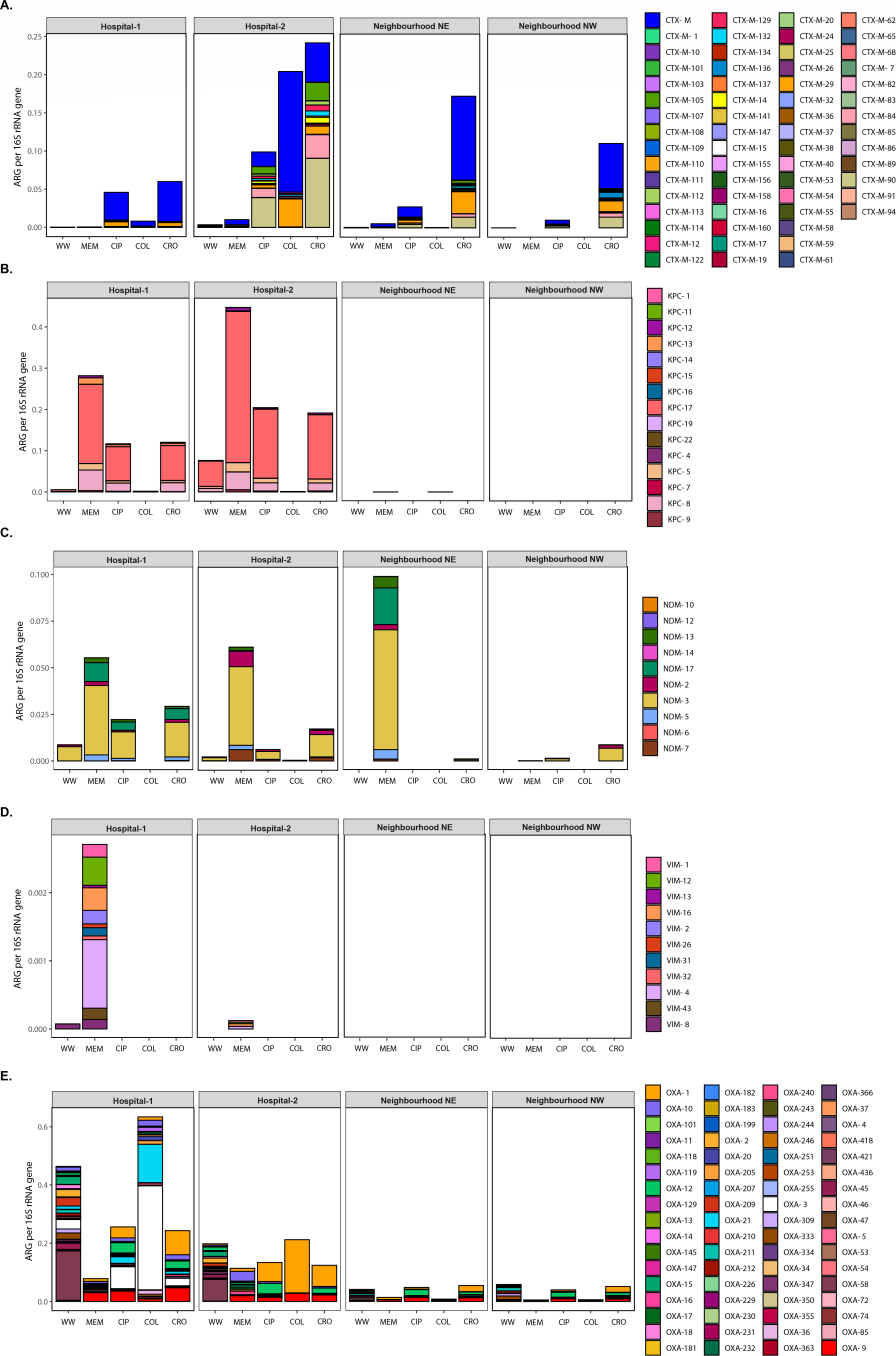
Normalized abundance of select β-lactamases found in the resistome of wastewater before and after culture enrichment on antibiotic-impregnated MacConkey agar. Normalized abundance is expressed as numbers of ARG per 16S rRNA gene for (**A**) CTX-M, (**B**) KPC, (**C**) NDM, (**D**) VIM, and (**E**) OXA, from raw and culture-enriched wastewater samples (MEM, CIP, COL, and CRO) collected from hospitals (Hospital-1 and Hospital-2) and neighborhoods (NE and NW). ARG variants are color-coded. In panel A, CTX-M allelic variants that were not classified as a specific variant during the read-based annotation step are clustered and color-coded in the same group. In panel E, for ease of illustration, only the 68 out of 152 variants that were found with a normalized abundance >0.0005 within the OXA group are plotted. WW: raw wastewater, MEM: meropenem, CIP: ciprofloxacin, COL: colistin, and CRO: ceftriaxone.

To characterize in greater detail the beta-lactamases that had the highest (i.e., CTX-M, KPC, NDM, and VIM) or lowest (i.e., OXA) EF after semi-selective culture enrichment, the 16S rRNA gene normalized ARG abundance was analyzed by ARG subtypes. We found 61 different CTX-M enzyme subtypes, where CTX-M-90, -29, and -84 genes were the most abundant, found across all sample locations and antibiotics tested (Fig. 5A). CTX-M-14 and CTX-M-15, emerging clinically important ESBL variants, were found in many conditions (i.e., before and after culture enrichment) across all sample locations (Data set S1). Fifteen KPC variants were detected across all resistomes analyzed. KPC-17 and -8 were the most abundant and found across all samples (Fig. 5B). However, 11 KPC variants were only identified after culture enrichment in one or more samples, predominantly those from hospitals (Fig. S9). Ten NDM variants were identified across all resistomes analyzed. NDM-3, -17, and -2 were the most abundant variants found across all samples (Fig. 5C). Importantly, all the NDM variants were only detected after culture-enrichment in one or more samples (Fig. S9). Eleven VIM variants were identified across all resistomes analyzed, all of them exclusively identified from hospitals. VIM-4 and -12 were the most abundant variants (Fig. 5B). Out of the 11 VIM variants, 10 were only identified after culture enrichment in hospital samples enriched with meropenem semi-selection (Fig. S9). Finally, we identified 152 OXA enzyme variants, where OXA-1, -3, and -9 were the most abundant across all samples (Fig. 5E). Half of the OXA variants (i.e., OXA-48, OXA-54, OXA-133, OXA-199, OXA-232, OXA-244, OXA-247, and OXA-436) belonging to OXA-23-like and OXA-48-like subfamilies (i.e., clinically important carbapenemases) were identified in cultured-enriched samples (Fig. S9).

### Composition profiles of ARGs in culture-enriched wastewater samples

**Fig 6:**
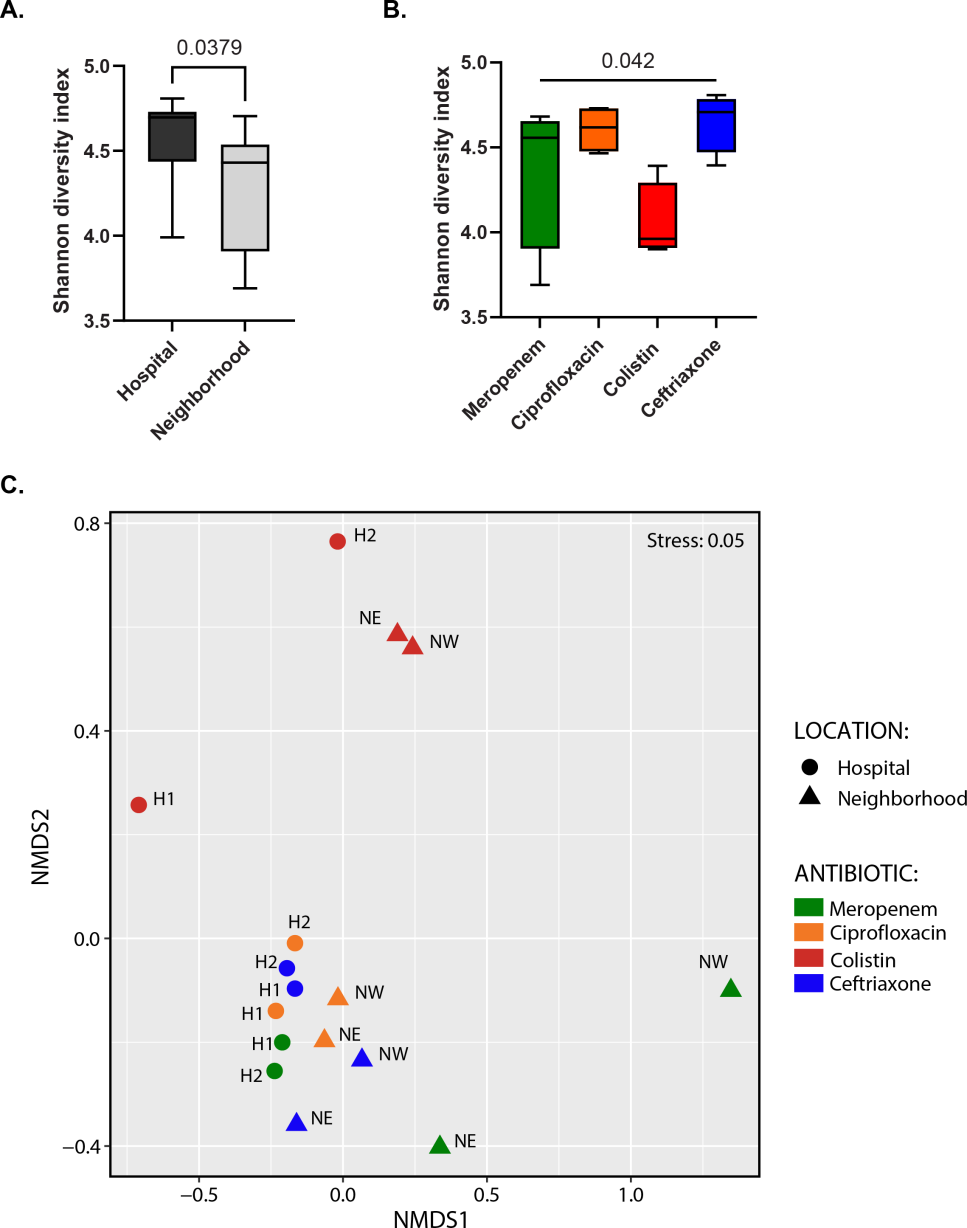
ARG subtype alpha- and beta-diversity of wastewater resistome after culture enrichment. Comparison of the ARG alpha-diversity measured using the Shannon Diversity index of the culture-enriched wastewater samples based on (**A**) sample location and (**B**) antibiotic used for selective culture enrichment as determined by Mann-Whitney test and Kruskal-Wallis test, respectively. (**C**) NMDS plot was done on the Bray-Curtis distances of ARG subtype profiles of the culture-enriched wastewater samples. Samples were shape-coded based on the type of location where the sample was collected [i.e., hospital (H1: Hospital-1 and H2: Hospital-2) or neighborhood (NE and NW)] and color-coded based on the antibiotic used for selection.

ARG diversity analysis of hospital and neighborhood culture-enriched wastewater was performed by measuring the Shannon diversity index (SDI). The median SDI for hospitals: 4.7 (IQR: 4.4–4.81) and neighborhoods: 4.43 (IQR: 3.91–4.54) was significantly different between locations regardless of enrichment condition (Fig. 6A, *P* < 0.05, Mann-Whitney test). We also analyzed the SDI to understand overall alpha-diversity across all culture-enriched conditions (Fig. 6B). Median SDI was the highest for wastewater enriched with ceftriaxone (SDI median 4.7, IQR: 4.4–4.7), followed by ciprofloxacin (SDI median 4.6, IQR: 4.4–4.7), meropenem (SDI median 4.5, IQR: 3.9–4.6), and colistin (SDI median 3.9, IQR: 3.9–4.2) (*P* < 0.05, Kruskal-Wallis test).

NMDS of wastewater ARG subtype diversity revealed an influence of sampling location (Fig. 6C), with similar results observed at the broader ARG type level (data not shown). Similarly, the NMDS ordination plot revealed a separation of resistomes based on the antibiotic used for selection. The distinction between hospitals and neighborhoods and between different antibiotics used for culture enrichment was further confirmed by ANOSIM (location type; R = 0.187, *P* = 0.011, antibiotic selection; R = 0.252, *P* = 0.004) and PERMANOVA tests (location type; R^2^ = 16, *P* = 0.015 and antibiotic selection; R^2^ = 40.03, *P* = 0.004). Beta-diversity analysis of the cultured-enriched ARGs visualized using NMDS showed a tight clustering by sampling location and antibiotic used for selection (Fig. S10). These differences were confirmed using the following complementary analyses: ANOSIM (location type; R = 0.345, *P* = 0.002 and antibiotic selection; R = 0.485, *P* = 0.0002) and PERMANOVA (location type; R^2^ = 20.8, *P* = 0.002 and antibiotic selection; R^2^ = 35.1, *P* = 0.006).

### Quantitative confirmation of differential detection of target ARG

We used qPCR to independently document the abundances of specific ARGs in raw wastewater. As beta-lactam resistance was the most prevalent ARG type that was identified (57.6%), we quantified target beta-lactamase genes in raw wastewater samples relative to the total bacterial load (i.e., 16S rRNA gene abundance). Using qPCR, we established that *bla*_CTX-M_, *bla*_KPC_, and *bla*_NDM_ were 9.6-fold, 4,066-fold, and 5,924-fold higher in raw hospital wastewater compared with raw neighborhood wastewater (Fig. S11A). These studies confirmed that metagenomic observations (Fig. S11A and B), where there was a strong correlation between metagenomic and qPCR measurements (i.e., *bla*_CTX-M_ (R^2^ = 0.997, *P* = 0.003), *bla*_KPC_ (R^2^ = 0.99, *P* = 0.001), and *bla*_NDM_ (R^2^ = 0.955, *P* = 0.045)). We likewise quantified the presence of *vanA* as a surrogate for vancomycin-resistant *Enterococcus* (VRE), as well as total *C. difficile* and one of its enterotoxin genes *tcdA,* in raw wastewater. We found that *vanA*, total *C. difficile* and *tcdA* relative abundance were 14.5-fold, 11.8-fold, and 19.7-fold higher in hospitals than in neighborhood wastewater (Fig. S11C). Finally, since the culture enrichment of wastewater samples was performed on MacConkey agar plates that select against gram-positive bacteria, we used qPCR to confirm that *vanA* values were lower in culture-enriched samples relative to raw wastewater (Fig. S11D). Gene abundances for *vanA* determined by qPCR correlated with metagenomic results (Fig. S11E and F).

### Comparison of ARGs in wastewater with AMR in clinical isolates

We compared the ARGs in wastewater with the burden of antibiotic resistance clinically detected in hospitals and across the community, compiled in annual antibiograms. Significant differences in AMR rates among key clinical pathogens, including *E. coli*, *K. pneumoniae* complex, *Enterobacter cloacae* complex, and *P. aeruginosa,* were observed between hospitals and the community (Table S3). In general, Hospital 1 had higher rates of resistance to key drugs (i.e., ceftriaxone, ciprofloxacin, cefazolin, ampicillin, amoxicillin-clavulanic acid, piperacillin-tazobactam, and ceftazidime) than Hospital 2 and much higher than the overall community (Table S3). These results are in accordance with the wastewater data, where hospital samples had higher abundance and diversity of ARGs compared with community samples.

## DISCUSSION

Interest in wastewater-based surveillance of antimicrobial resistance has been progressively growing. It provides an integrated view of ARG burden across a population that is objective, comprehensive, and inclusive. Furthermore, it can be performed longitudinally and at a tiny fraction of the cost of serial active surveillance to monitor for changes in the prevalence of specific ARGs that in certain pathogens (e.g., members of the *Enterobacterales* or *P. aeruginosa*) lead to healthcare-associated outbreaks (43, 44). Despite the great potential of this technology, its application to hospitals has been limited to date, and this is where this study comparing two tertiary-care hospitals with two communities is particularly relevant. Hospitals are where society’s most ill and vulnerable are cohorted together to receive care. As a consequence, patients in hospitals are simultaneously the most susceptible to acquiring infection and experiencing adverse outcomes as a result of that infection. Given that 37%–44% of admitted patients receive antibiotics during their hospital stay (40, 41), the selection pressure for ARO colonization and subsequent infection is particularly intense. This presents an opportunity for dynamic surveillance mechanisms to monitor for changing prevalence of ARGs and (in the future) the prevalence of pathogens that carry these ARGs. Wastewater testing can meet the need to augment antimicrobial stewardship and infection prevention and control programs (40, 41, 45, 46). For example, here, we have shown how AMR surveillance through wastewater can complement more traditional culture-based programs such as annual antibiograms by providing AMR profiles through pooled sampling (43). However, WBS can do so faster (in near real time), cheaper, and more comprehensively. This need and opportunity are underscored by our observation that the resistome in hospital populations differs markedly from the surrounding community, with higher levels of ARG from notable nosocomial pathogens. However, hospitals (and neighborhoods) serve different communities and have different clinical focuses—and as such, have their own resistomes—and accordingly, inclusion of more than one site provides greater insight. Indeed, significant differences in the resistomes between the two hospitals were observed herein and correlated with those observed through clinically identified pathogens.

Our results expand on early approaches that have been used to analyze antimicrobial resistance in hospital wastewater (47–49). For example, Buelow and collaborators (50) studied the resistomes of specific hospitals and compared these with urban wastewater but identified only a few ARGs using a high-throughput qPCR (50). Other studies have focused on the characterization of the diversity of specific ARGs (e.g., ESBLs and KPCs) in hospital wastewater exclusively with culture-dependent tools (51, 52). Carbapenemase-producing organisms have been identified from wastewater treatment plants specific to hospitals (53) and across a larger array of monitored sites irrespective of hospital contributions (54). Early metagenomic approaches using hospital wastewater have been described (46, 55–58); however, no study has introduced selective culture-enrichment to more comprehensively uncover resistomes in community or hospital wastewater samples. Our comparison of resistomes from different hospitals and neighborhoods increases the robustness of these observations.

We identified the presence of ARGs encoding resistance to a range of antibiotics, including beta-lactam, multidrug, fluoroquinolone, MLS, aminoglycoside, tetracycline, glycopeptide, diaminopyrimidine, phenicol, fosfomycin, and others, in diverse wastewater samples. The most abundant and diverse ARG types detected confer resistance to beta-lactam antibiotics and were found in both hospital and neighborhood wastewater. This is influenced by the high diversity of gene variants within the beta-lactam category in the databases used (42). We found that the alpha diversity of ARGs after culture enrichment was significantly higher in hospital samples compared with neighborhoods, highlighting the ability of this approach to uncover more of the resistome. Similar to our results, a previous study showed that hospital wastewater had a higher normalized abundance of ARGs than the other sample types (e.g., urban wastewater), particularly when comparing ARG types associated with beta-lactam and vancomycin resistance (~15-fold and ~175-fold higher in hospital wastewater, respectively) (50). These results are also supported by previous studies that have demonstrated that hospital wastewater contains richer and more abundant ARGs than municipal wastewater, which is proposed to relate to a higher burden of ARO colonization among hospitalized individuals (56, 59). Within the “environmental” One Health lens, the high concentrations of antibiotics in hospitals relative to municipal populations create greater selective pressure on the microbiota, driving resistance (57). Hospital wastewater therefore serves as a particular hotspot for ARG dispersal into watersheds and the broader environment, potentially reaching animals and humans (55, 60–62). Cai et al. (63) performed weekly wastewater surveillance of a single ~250-bed hospital in Shantou, China, for ARG using metagenomics over a period of several months. They compared their wastewater-derived data with pathogens isolated from patients and used network analysis to demonstrate strong correlations with many ARG types and recovered clinical pathogens, similar to what we have observed herein (63). This highlights the importance of comparative studies that assess the link between organisms isolated from wastewater and the resistance genes found in the samples (63–65).

Where this study differs relative to prior work is its inclusion of a limited suite of semi-selective culture-enrichment conditions to improve the detection of clinically important, but rare ARGs. Although metagenomic sequencing is a robust approach for ARG identification and characterization relative to traditional targeted studies, the sequencing depth that is required for ARG detection is determined by the relative abundance of the target gene in the overall sample, which can be exceptionally low (66, 67). Coupling limited selective culture-enrichment of wastewater with lower-resolution metagenomic sequencing allowed us to detect many additional ARG subtypes (including clinically important KPCs, NDMs, and VIM metallo-beta-lactamases) (Fig. S9) that were not detected in higher-resolution metagenomes of raw wastewater (Table 1S). Therefore, selective culture-enrichment could potentially identify a hidden reservoir of unrecognized important ARGs that, in certain pathogens, lead to nosocomial outbreaks, allowing preventive infection prevention and control and stewardship strategies to be judiciously applied. Observations of VIM genes following selective enrichment with hospital wastewater (Fig. 5D) are especially interesting, given its association with recurrent outbreaks unique to our local hospitals with significant associated morbidity and mortality (68). Such an approach can manifest in cost savings—particularly relevant for high-volume longitudinal monitoring programs. Interestingly, increased detection of these targets (i.e., *bla*_KPC_, *bla*_NDM_, and *bla*_VIM_) occurred using both the beta-lactam enrichment conditions: meropenem and ceftriaxone, in addition to ciprofloxacin, likely relating to the co-location of ARG on transmissible mobile elements in resistant pathogens (10). The exception to antibiotic enrichment was colistin, which negatively selected against carbapenemases and only modestly enriched for the emerging *mcr*-1 (69), which was exceedingly infrequent in the raw and cultured-enriched samples from neighborhoods (consistent with its exceptionally uncommon use across our health region). This suggests potential for a broader screening strategy, with additional selective agents that may be needed for comprehensive wastewater surveillance of AMR using this approach.

Although most of the samples clustered by the selective antimicrobial agent used, in some instances (i.e., neighborhood sample culture in meropenem), the enrichment condition did not explain the variance of the resistome. In some treatments, we observed unique ARGs that were significantly associated with the sample in comparison to other locations using the same enrichment conditions, revealing a distinct resistome profile driven by location-specific factors rather than the selective agent alone. These factors may relate to differences in the population make-up of those locations. For example, NE includes an almost 2-fold higher proportion of residents who had previously immigrated to Canada from another country (70). Although the types of ARGs enriched depended on individual conditions, the meropenem treatment provided the most diversity and was most likely to identify rare carbapenemases (i.e., *bla*_KPC_, *bla*_NDM_, and *bla*_VIM_) when assessed across all sites. At the ARG type level, we identified a bicyclomycin ARG, uniquely in the culture-enriched wastewater samples. Resistance to bicyclomycin is mediated by a transmembrane protein that excludes it from penetrating the cell (71), and it has been found elsewhere in *P. aeruginosa* strains responsible for outbreaks (72).

A common critique of metagenomic studies is the inability to distinguish between live and dead organisms. This is particularly relevant if infection control measures are to be implemented based on wastewater metagenomics. However, culture enrichment mitigates this. Few other studies have used a similar approach, and they have only focused on AMR in influent and effluent municipal wastewater. Zhang and collaborators (34) assessed the resistome through culture-enriched phenotypic metagenomics in municipal wastewater treatment plants and their receiving river samples, the latter being critical for evaluating the risk of ARG dissemination into ecosystems. This study detected a greater diversity of ARGs when coupling culture enrichment and metagenomic analysis than direct metagenomic sequencing alone, with a particular focus on carbapenemases and ESBLs (34). Marano and collaborators (39) used culture enrichment under copiotrophic conditions to enrich and determine the microbiome and resistomes in soil that was irrigated with treated wastewater. They observed cephalosporin- and carbapenem-resistant *Enterobacterales* only after culture enrichment, not in raw soil samples (39). Other alternatives to culture enrichment prior to metagenomic analysis consist of using commercially available AMR target enrichment NGS panels (43). These approaches offer high sensitivity for the detection of low-abundance AMR genes via probe-based hybridization or PCR enrichment with a faster turnaround as no culturing step is involved (73), but this approach is limited to the targets included in the panels; therefore, it may underestimate the discovery of novel or poorly characterized AMR genes or sequence related to mobility elements that contribute to the spread of AMR. The approach in this study overcomes many of the limitations, allowing lower-abundance genes and organisms to be sequenced without resorting to extremely deep and very expensive shotgun sequencing strategies.

Targeted surveillance of specific clinically relevant ARGs serves to complement metagenomic assessments. Indeed, others have suggested that this approach has increased sensitivity relative to shotgun metagenomics (38). Adapting targeted qPCR, we confirmed marked increases in clinically relevant ARGs in hospital wastewater relative to community samples, including genes encoding beta-lactamases that are carried by nosocomial gram negatives (i.e., CTX-M ESBLs, KPCs, and NDM) and gram-positive pathogens (i.e., total *C. difficile* and *tcdA*, and *vanA* from VRE).

We acknowledge several limitations of this study. We specifically used a lower sequencing target depth in culture enrichment samples (sequencing output target of ~6 gigabases vs ~10 gigabases for raw wastewater samples) in order to assess if this process could manifest in cost savings for the identification of rare ARGs. The use of selective culture enrichment, designed to increase the identification of any specific target, will conversely mask others that cannot grow under those conditions. As our goal was to assess for rare gram-negative ARGs, which are presumed to be in ARO that colonize only a small fraction of the population and exist as minor constituents within their resident microbiota, we enriched with MacConkey agar and appropriate antibiotics to select for these mechanisms, rather than focusing on longitudinal differences across sites. This approach is reflected in our limited sample size. Importantly, we observed that there were not great differences in AMR gene detections in each of the culture-enriched conditions—and that future work that augmented the raw resistome with only one enrichment condition (i.e., meropenem, which demonstrated the greatest diversity) would be sufficient. In doing so, we selected against other clinically relevant nosocomial pathogens (i.e., VRE and other gram positives), which were nevertheless readily identified in raw samples, owing to their increased prevalence and abundance in colonized/infected individuals. This study did not assess the relationship between antibiotics released into hospital wastewater and the abundance of ARGs found in those samples. Only a few studies have examined this relationship, where correlations between antibiotic concentrations (as a proxy for antibiotic use) and ARG in wastewater were demonstrated (55, 74). Finally, our approach neither allowed the taxonomic identification of those organisms carrying ARGs of interest nor did we assess the role of mobile genetic elements in the distribution of antimicrobial resistance in hospital and community wastewater. Although not all organisms carrying ARGs may pose direct threats to human populations, they may serve as reservoirs from which these genes are spread to pathogens. Although metagenomic data alone do not confirm phenotypic resistance, the use of selective media was intended to enrich for organisms exhibiting resistance to these antibiotics. Future studies should include VRE and C. difficile. By adopting steps to allow for the identification and characterization of organisms carrying these genes (e.g., isolation of individual isolates from wastewater or alternative metagenomic approaches such as *de novo* assembly of metagenomes that utilize genome linkage approaches (e.g., Hi-C) that could enable taxonomic inferences from sequencing data) (75). Together, these could enable the reconstruction of metagenome-assembled genomes to analyze the presence of potentially pathogenic bacteria harboring ARGs (76). We note, however, that an additional advantage of culture-based enrichment is the potential to retrospectively revisit archived stocks to isolate individual pathogens carrying ARGs of interest for greater characterization through whole genome analysis.

Here, we were able to demonstrate using multiple independent sampling locations and complementary modalities (i.e., metagenomics with and without culture enrichment, and targeted qPCR) that the resistome of hospitals significantly differs from communities and is notable for prevalent beta-lactam resistance in gram negatives as well as notable nosocomial pathogens, including VRE and *C. difficile*. By adopting simple, semi-selective culture enrichment conditions, we were able to identify ultra-rare and clinically relevant ARG, such as NDM, VIM, and KPC, while simultaneously reducing the costs associated with sequencing. Although all four conditions increased clinically important ARG detection, this results in a significant increase in costs relative to monitoring a single condition. Future studies using only meropenem are likely to provide the greatest additional information to raw metagenomic data and are well suited for the next step, which includes longitudinal monitoring. Wastewater-based surveillance is a potentially important and powerful tool that warrants exploration as a tool to augment hospital-based infection control and antimicrobial stewardship programs.

## MATERIALS AND METHODS

### Setting and wastewater collection methods

Composite wastewater samples (one sample per location) were collected from two hospitals (Hospital 1 and Hospital 2) and two nearby neighborhoods (NE and NW) (Fig. S1) in the summer of 2021 in Calgary, Alberta, Canada. ISCO 6712 portable samplers (Lincoln, Nebraska) were installed at the designated manholes and programmed to collect and store 24-h composite wastewater samples as follows: 100 mL of wastewater every 15 min, for a total of 96 pooled samples over a period of 24 h. The same time and date of collection (June 14, 2021) was used for all four sites. Hospital sampling locations were chosen to provide comprehensive and exclusive coverage of each hospital’s wastewater system (20). These two hospitals encompass 47% of regional tertiary-care adult beds in the City of Calgary (population 1.3 million): Hospital 1 (Northeast Calgary, ~600 inpatient beds) and Hospital 2 (Southwest Calgary, ~650 inpatient beds). Neighborhood locations were chosen based on ease of access and similar sizes of population served by each sewershed (i.e., NE: 44,839 and NW: 46,008)—where population size was determined based on the number of service connections. The two neighborhoods also capture populations of diverse socioeconomic backgrounds (21). Federal census data from 2021 reveals that NE has a higher average household size of 3.78 vs NW 2.72 individuals; a percentage of residents identifying as belonging to a visible minority of 81.1% vs 35.6%, and an average of household family income of CAD $36,809 vs $68,228 (21). In general, Hospital 1 provides the majority of routine care to residents of Neighborhood 1, and Hospital 2 provides disproportionate routine care to residents of Neighborhood 2. In addition to general medical, surgical, and intensive care programs at each site—each hospital hosts unique medical and surgical programs that serve the entire regional area. Hospital 1 includes the regional vascular surgery program and has a large dialysis and hematology/oncology program, whereas Hospital 2 includes the regional urology and ophthalmology programs. Wastewater samples were time-weighted, whereby autosamplers were programmed to collect 100 mL every 15 min, for a total of 96 pooled samples over a period of 24 h. Samples were collected and transported on ice by City of Calgary staff using a standardized protocol (20, 21). This project was approved by the Conjoint Regional Health Ethics Board (REB20-1252).

### Wastewater processing and culture enrichment

Isolation of biomass from wastewater followed steps adapted from a published protocol (77). Two aliquots of 40 mL were taken from each composite wastewater sample after thorough mixing. Samples were centrifuged at 4,200 rpm for 20 min at 4°C. The supernatant from each was carefully removed and discarded, and the pellets were combined. The total volume of pellets recovered was recorded and used for further calculations. All processed pellet samples (*n* = 4) were either stored at −20°C before DNA extraction or were immediately used to inoculate semi-selective media for culture enrichment (*n* = 16).

To increase the detection of rare ARGs from gram negatives, 50 µL of each fresh pellet was plated onto MacConkey agar plates supplemented with either 8 µg/mL meropenem (MEM), 4 µg/mL ciprofloxacin (CIP), 8 µg/mL ceftriaxone (CRO), or 2 µg/mL colistin (COL) (Table S2). These antibiotics were chosen to represent a range of clinically relevant resistance phenotypes, thereby promoting the growth of ARO and enhancing the detection of associated ARGs through metagenomic analysis. Plates were incubated overnight for 14 h at 37°C. The resulting biomass was collected by adding 1 mL of UltraPure DNase/RNase-Free Distilled Water (Invitrogen) to plates and physically scraping to collect the resultant biomass. The total volume of the culture-enriched product was recorded and used for further calculations. All the processed culture-enriched wastewater samples were stored at −20°C before DNA extraction.

### DNA extraction, library preparation, and shotgun metagenomic sequencing

Aliquots of 250 µL of either the raw or culture-enriched wastewater samples were used for total genomic DNA extraction using the DNeasy PowerSoil Pro DNA Isolation Kit (Qiagen) according to the manufacturer’s recommendations. A blank extraction control was included for every processed sample batch to ensure no contamination occurred. DNA concentration was measured using a Qubit fluorometer (Thermo Fisher Scientific). Library preparation and shotgun metagenomic sequencing were performed by Novogene Corporation Inc., California, USA. Paired-end sequencing (2 × 150 bp) was performed on the Illumina Novaseq 6000 PE150 platform with a sequencing output target of ~10 gigabases (~33M paired reads/sample) for the four raw wastewater and ~6 gigabases (~20M paired reads/sample) for 16 culture-enriched samples (Table S1). The median final outputs were 15.2 (IQR = 12.5–17.6 gigabases) and 7.9 (IQR = 7.3–8.6 gigabases) for raw wastewater and culture-enriched samples, respectively (Table S1).

### Bioinformatics workflow

Illumina raw reads were initially filtered by Novogene to remove adapters, low-quality reads, and reads containing *N* > 10% (i.e., N representing bases that cannot be determined). Base qualities and adapter contamination were then double-checked using FastQC (v.0.11.9) (78), and reads were further trimmed using Trimmomatic (v0.39) (79). DeepARG-SS pipeline v1.0.2 was used for gene annotation, in which it aligns reads compiled from multiple public databases (i.e., CARD, ARDB, UniProt for ARGs, and Greengenes for 16S rRNA genes) (42). DeepARG-SS pipeline v1.0.2 outputs were subject to further analysis to calculate copies of both ARGs and 16S rRNA gene and normalized ARG abundance for each ARG subtype homologs (e.g., OXA-1, -2, etc.) according to a customized workflow written in Python (v3.5.3) and described in the Supplemental Material.

### Objective quantification of targeted genes

Targeted qPCR was performed to quantify the different ARGs of interest (i.e., *bla*_CTX-M_, *bla*_KPC_, *bla*_NDM_, and *vanA*) in raw wastewater and culture-enriched samples and to validate the metagenomic data. For each gene assay, 10 µL reactions contained 5 µL of TaqMan Fast Advanced Master Mix (Applied Biosystems), 0.5 µL of TaqMan Gene Expression assay (Thermo Fisher Scientific) (Table S4), and 4 µL of the template DNA. For all assays, the qPCR program reaction consisted of an initial step at 50°C for 2 min, then a step at 95°C for 2 min, followed by 40 cycles of 95°C for 1 s, 60°C for 20 s. A 9-fold dilution series of double-stranded DNA fragments (Integrated DNA Technologies (IDT), USA) was synthesized for each qPCR target assay (Table S5) and was run in triplicate on every 96-well PCR plate to produce standard curves that were in turn used to estimate absolute gene abundance. Workflows describing the absolute quantification of *C. difficile* and its toxin *tcdA*, and total bacterial load in wastewater are described in the supplemental material. All qPCR reactions were carried out using the QuantStudio 5 Real-time PCR systems (Applied Biosystems).

### Clinical data collection

As part of routine clinical care, antibiograms are created each year for individual hospitals and the overall city of Calgary using isolates recovered from individuals in each catchment (i.e., blood, sputum, urine, and wound cultures, etc.) with clinically evident infections (80). Only one isolate per patient is included, and only when sufficient isolates are collected are data reported. Pathogen identification and susceptibility testing are performed using standard procedures following CLSI guidelines (81).

### Statistical analysis

Alpha-diversity was calculated to estimate the ARG diversity from the metagenomic sequencing data using the Shannon Diversity index (SDI). SDI was compared between culture-enriched wastewater samples from hospitals (*n*  =  8) and neighborhoods (*n* = 8) using a Mann-Whitney test (α  =  0.05). The Kruskal-Wallis test was performed to test for differences in SDI across samples after culture enrichment based on the antibiotics used. Beta-diversity clustering plots (i.e., non-metric multidimensional scaling [NMDS]) were generated using the Bray-Curtis dissimilarities metrics of the ARG subtypes to visualize potential clustering patterns among samples. Analysis of similarity (ANOSIM) was conducted to determine the significance of dissimilarities between/among groups. Permutational multivariate analysis of variance (PERMANOVA) tests were performed to test differences in the Bray-Curtis distance matrices. Both ANOSIM and PERMANOVA were performed with permutations = 999 using the vegan package in R. Biplot analysis was performed to correlate species data (i.e., ARG subtypes) with the abovementioned NMDS ordination using the vegan package in R (82). The *P* value for biplot analysis was calculated using permutation (*n* = 999). A heatmap was generated for the selected ARGs of interest according to the results from biplot analysis in R. Associations between qPCR and metagenomic data from selected targets in raw wastewater (*bla*_CTX-M_, *bla*KPC, *bla*_NDM_, and *vanA*, *n* = 4), and cultured-enriched (*vanA*, *n* = 16) samples were generated using a linear model. Differences in proportions were assessed using the Fisher exact tests as appropriate for multiple groups. When appropriate, *P*-values were adjusted for multiple comparisons by using Benjamini–Hochberg *P*-value correction for multiple testing. All data analyses and visualizations were performed using GraphPad Prism-10 software (La Jolla, CA) and in R (V4.0.4).

## Data Availability

The metagenomic data generated in this study have been deposited on the National Center for Biotechnology Information (NCBI) Sequence Read Archive (SRA) repository under the under the BioProject ID PRJNA947333. All source data for the results reported in this study are provided in the Supplementary file.

## SUPPLEMENTAL MATERIAL

Data Set S1 mbio.01672-25-s0001.xlsx ARG data, sheets 1 to 3.

Supplemental Material mbio.01672-25-s0002.docx Supplemental methods, Tables S1 to S5, and Fig. S1 to S11.

ASM does not own the copyrights to Supplemental Material that may be linked to, or accessed through, an article. The authors have granted ASM a non-exclusive, world-wide license to publish the Supplemental Material files. Please contact the corresponding author directly for reuse.
