# Supplementary material for: Metagenomic analysis after selective culture enrichment of hospital and community wastewater enhances antimicrobial resistance gene detection
Source: mBio. 2025 Jul 31;16(9):e01672-25. doi: 10.1128/mbio.01672-25 (PMC12421813; doi:10.1128/mbio.01672-25)
Supplement: Supplemental Material — Supplemental methods, Tables S1 to S5, and Fig. S1 to S11. [file mbio.01672-25-s0002.docx]

**Supplemental Material for**

**Metagenomic analysis after selective culture enrichment of hospital and community wastewater enhances antimicrobial resistance gene detection**

Nicole Acosta^a^, Jangwoo Lee^a^, Maria A. Bautista^b^, Srijak Bhatnagar^c^, Carmen Li^b^, Barbara J. Waddell^a^, Emily Au^a^, Puja Pradhan^a,b^, Rhonda G. Clark^b^, Jon Meddings^d^, Gopal Achari^e^, Johann D. Pitout^a,f^, John Conly^a,d,f-i^, Kevin Frankowski^j^, Casey R.J. Hubert^b^, Michael D. Parkins ^a,d,i#^

**SUPPLEMENTARY METHODS**

**Bioinformatics workflow**

DeepARG-SS pipeline v2.0 outputs were subjected to further analysis, to calculate copy of both ARGs and 16S rRNA gene (E1) and normalized ARG abundance (E2) for each ARG subtype homologues (e.g., OXA-1, -2, etc.) according to a customized workflow written in Python (v3.5.3).

(E1)

$$Copy= \frac{Number of reads x Read length}{Reference length}$$

$$Normalized ARG abudance= \frac{Copy for ARG}{Copy for 16S rRNA gene}$$

(E2)

where number of reads is the number of ARG reads identified to a specific ARG; read length is the sequence length (bp) of the Illumina reads (i.e., 150 bp); reference length is the sequence length of the corresponding target ARG sequence referring to DeepARG-DB v2.0 database ^1^; Number of 16S rRNA gene is the number of the 16S rRNA gene sequence identified in each sample; and the 16S rRNA gene length is the sequence length of 16S rRNA gene (i.e., 1432 bp) ^2^. All downstream analyses were performed using 16S rRNA gene-normalized ARG abundance referring to other studies ^3-5^. Identified ARG subtypes were categorized and aggregated by ARG types which refers to the antimicrobial agents to which each gene confers resistance (Fig. 1A). The workflow using an example dataset is available on GitHub (<https://github.com/myjackson>). The raw reads are available in the National Center for Biotechnology Information (NCBI) Sequence Read Archive (SRA) repository, under the BioProject ID PRJNA947333 (Reviewer link https://dataview.ncbi.nlm.nih.gov/object/PRJNA947333?reviewer=ec5mbch891ssoq7hivkktev4db). *The data will be released to the public when the manuscript is formally accepted for publication.*

**qPCR assays**

For total burden of *Clostridioides difficile* and its toxin *tcdA*, each 10 µl reaction contained 5 µl of TaqMan® Fast Advanced Master Mix (Applied Biosystems), 0.5 µl of a mix of forward and reverse primers, 0.5 µl of probe (Supplementary Table 2); and 4 µl of the DNA template. For both *C. difficile* 16S rRNA and *tcdA* qPCR assays, the qPCR program reaction consisted of an initial step at 50˚C for 2 min, then a step at 95˚C for 2 min followed by 50 cycles of 95˚C for 1 s, 56˚C for 20 s. A 7-fold dilution series of double-stranded DNA fragments (Integrated DNA Technologies (IDT), USA) was synthesized and used (Supplementary Table 3) and were run in triplicate on every 96-well PCR plate to produce standard curves and used to estimate the absolute quantity.

For establishing total bacterial load, we measured 16S rRNA gene/ml of wastewater. Each 10 µl reaction contains 5 µl of TaqMan® Fast Advanced Master Mix (Applied Biosystems), 0.5 µl of a mix of forward and reverse primers, 0.5 µl of probe (Supplementary Table2); and 2.5 µl of the DNA template. The qPCR program reaction consisted of an initial step at 50˚C for 2 min, then a step at 95˚C for 2 min followed by 40 cycles of 95˚C for 1 s, 62 ˚C for 20 s. A 9-fold dilution series of *P. aeruginosa* PA01 genomic DNA (62.5 to 1.6 X 10^-4^ ng/µl) was used as standard curve. Data were converted from DNA concentration (ng/µl) to 16S rRNA gene number as previously described by Zhao et al ^6^. The number of 16S gene in a given reaction was multiplied by the total number of nanograms in the entire sample DNA prep and total eluted volume after DNA extraction and then divided by the volume of wastewater used for DNA extraction to give an absolute abundance of 16S gene /ml of wastewater. All samples were assayed in duplicate, and all runs contained non-template control. All qPCR reactions were carried out using the QuantStudio 5 Real-time PCR systems (Applied Biosystems).

**SUPPLEMENTARY TABLES**

**Table S1.** Overview of the paired-end sequencing (2 × 150 bp) sequencing results obtained by Illumina Novaseq 6000 PE150 platform.

| Sample ID | Sequencing output (Gb)^*^ | Raw reads | Location | Type^α^ |
| --- | --- | --- | --- | --- |
| NA_1 | 17.5 | 116,338,888 | Hospital-1 | raw wastewater |
| NA_2 | 17.7 | 118,220,046 | Hospital-2 | raw wastewater |
| NA_3 | 8.6 | 57,521,818 | Hospital-1 | culture-enriched-MEM |
| NA_4 | 7.4 | 49,324,432 | Hospital-1 | culture-enriched-CIP |
| NA_5 | 8.6 | 57,428,492 | Hospital-1 | culture-enriched-CRO |
| NA_6 | 7.4 | 49,060,778 | Hospital-1 | culture-enriched-COL |
| NA_7 | 7.5 | 49,983,644 | Hospital-2 | culture-enriched-MEM |
| NA_8 | 8 | 53,320,788 | Hospital-2 | culture-enriched-CIP |
| NA_9 | 9 | 59,970,976 | Hospital-2 | culture-enriched-CRO |
| NA_10 | 6.1 | 40,860,274 | Hospital-2 | culture-enriched-COL |
| NA_11 | 11.1 | 74,092,986 | NE | raw wastewater |
| NA_12 | 12.9 | 85,855,692 | NW | raw wastewater |
| NA_13 | 8.6 | 57,157,698 | NE | culture-enriched-MEM |
| NA_14 | 7.7 | 51,630,126 | NE | culture-enriched-CIP |
| NA_15 | 8.3 | 55,454,866 | NE | culture-enriched-CRO |
| NA_16 | 6 | 39,933,826 | NE | culture-enriched-COL |
| NA_17 | 11.6 | 77,036,096 | NW | culture-enriched-MEM |
| NA_18 | 6.8 | 45,107,562 | NW | culture-enriched-CIP |
| NA_19 | 8 | 53,285,664 | NW | culture-enriched-CRO |
| NA_20 | 7.1 | 47,359,372 | NW | culture-enriched-COL |

*Sequencing output = raw reads x sequencing length (i.e., 150)

^α^MEM: meropenem, CIP: ciprofloxacin, COL: colistin and CRO: ceftriaxone.

**Table S2.** EUCAST and CLSI minimum inhibitory concentration breakpoints for nosocomial pathogens for antibiotics used herein.

|  | MIC Breakpoint (µg/ml) | | | | | | | |
| --- | --- | --- | --- | --- | --- | --- | --- | --- |
| Pathogen | EUCAST | CLSI | EUCAST | CLSI | EUCAST | CLSI | EUCAST | CLSI |
|  | Ceftriaxone | | Meropenem | | Ciprofloxacin | | Colistin | |
| *Pseudomonas aeruginosa* | - | - | 2 (S≤), 8 (R>) | 2 (S≤), 8 (R>) | 0.001 (S≤), 0.5(R>) | 0.5 (S≤), 2(R>) | 4 (S≤), 4 (R>) | 4 (R>) |
| *Escherichia coli* | 1 (S≤), 2(R>) | 1 (S≤), 4(R>) | 2 (S≤), 8 (R>) | 1 (S≤), 4 (R>) | 0.25 (S≤), 0.5(R>) | 0.25 (S≤), 1(R>) | 2 (S≤), 2 (R>) | 4 (R>) |
| *Klebsiella pneumoniae* | 1 (S≤), 2(R>) | 1 (S≤), 4(R>) | 2 (S≤), 8 (R>) | 1 (S≤), 4 (R>) | 0.25 (S≤), 0.5(R>) | 0.25 (S≤), 1(R>) | 2 (S≤), 2 (R>) | 4 (R>) |
| Concentration used in study | 8 | | 8 | | 4 | | 2 | |

EUCAST: European Committee on Antimicrobial Susceptibility Testing^7^, CLSI: Clinical and Laboratory Standards Institute^8^, MIC: minimum inhibitory concentration, S= susceptible, R= resistant.

**Table S3.** Susceptibility rates of key Gram-negative pathogens from across the Calgary Health Region and two hospitals included in the study derived from Annual Antibiograms^9^.

| **Organisms** | **Total Isolates** | **Ampicillin** | **Amoxicillin-Clavulinic Acid** | **Piperacillin-Tazobactam** | **Ceftriaxone** | **TMP-SMX** | **Ciprofloxacin** | **Nitrofurantoin** | **Tobramycin** | **Meropenem** | **Doxycycline** | **Cefazolin** | **Ceftazidime** |
| --- | --- | --- | --- | --- | --- | --- | --- | --- | --- | --- | --- | --- | --- |
| ***Escherichia coli*** |  |  |  |  |  |  |  |  |  |  |  |  |  |
| Community | 17855 | 64% | 89% | 92% | 92% | 82% | 73% | 99% | 93% |  |  | 87% |  |
| PLC | 241 | 51% | 78% | 78% | 78% | 73% | 63% | 98% | 89% |  |  | 66% |  |
| RGH | 304 | 57% | 85% | 85% | 85% | 83% | 67% | 98% | 93% |  |  | 77% |  |
| **P-value*** |  | <0.001 | <0.001 | <0.001 | <0.001 | 0.002 | <0.001 | 0.049 | 0.051 |  |  | <0.001 |  |
| ***Enterobacter cloacae* complex** |  |  |  |  |  |  |  |  |  |  |  |  |  |
| Community | 445 |  |  |  |  | 92% | 93% | 34% | 99% | 100% |  |  |  |
| PLC | 51 |  |  |  |  | 92% | 88% |  | 96% | 100% |  |  |  |
| RGH | 41 |  |  |  |  | 93% | 85% |  | 95% | 98% |  |  |  |
| **P-value*** |  |  |  |  |  | 1 | 0.112 |  | 0.042 |  |  |  |  |
| ***Klebsiella pneumoniae* complex** |  |  |  |  |  |  |  |  |  |  |  |  |  |
| Community | 2172 |  | 95% | 96% | 96% | 92% | 89% | 33% | 98% |  |  | 95% |  |
| PLC | 68 |  | 93% | 93% | 93% | 93% | 88% | 45% | 99% |  |  | 88% |  |
| RGH | 90 |  | 96% | 96% | 96% | 92% | 90% | 45% | 98% |  |  | 91% |  |
| **P-value*** |  |  | 0.613 | 0.315 | 0.315 | 1 | 0.938 | 0.010 | 0.893 |  |  | 0.015 |  |
| ***Pseudomonas aeruginosa*** |  |  |  |  |  |  |  |  |  |  |  |  |  |
| Community | 464 |  |  | 97% |  |  | 83% |  | 97% | 93% |  |  | 98% |
| PLC | 78 |  |  | 92% |  |  | 87% |  | 97% | 87% |  |  | 92% |
| RGH | 97 |  |  | 95% |  |  | 89% |  | 100% | 94% |  |  | 95% |
| **P-value*** |  |  |  | 0.094 |  |  | 0.316 |  |  | 0.182 |  |  | 0.007 |

* Fisher's exact test was calculated to compare the proportion of resistant isolates across sites.

TMP-SMX: Trimethoprim-sulfamethoxazole.

**Table S4.** qPCR assays used for ARGs, *Clostridioides difficile* and total bacterial load quantification of wastewater samples.

| Assay | Assay ID or primers/probe name | Sequence (5’ – 3’) | Reference |
| --- | --- | --- | --- |
| *bla*_CTX-M_ | Ba04646149_s1 | TaqMan Gene Expression assay | (Thermo Fisher Scientific) |
| *bla*_KPC_ | Ba04646152_s1 | TaqMan Gene Expression assay |  |
| *bla*_NDM_ | Ba04931076_s1 | TaqMan Gene Expression assay |  |
| *vanA* | Ba04646147_s1 | TaqMan Gene Expression assay |  |
| *C. difficile* 16S rRNA gene | Forward primer | GCAAGTTGAGCGATTTACTTCGGT | ^10^ |
|  | Fluorescent probe | FAM/TGCCTCTCAAATATATTATCCCGTATTAG/MGBBNFQ |  |
|  | Reverse primer | GTACTGGCTCACCTTTGATATTYAAGAG |  |
| *C. difficile* *tcdA* | Forward primer | CAGTCGGATTGCAAGTAATTGACAAT |  |
|  | Fluorescent probe | VIC/TTGAGATGATAGCAGTGTCAGGATTG/MGBBNFQ |  |
|  | Reverse primer | AGTAGTATCTACTACCATTAACAGTCTGC |  |
| 16S rRNA Total bacterial load | Forward primer | TCCTACGGGAGGCAGCAGT | ^11^ |
|  | Fluorescent probe | FAM/CGTATTACCGCGGCTGCTGGCAC/MGBNFQ |  |
|  | Reverse primer | GGACTACCAGGGTATCTAATCCTGTT |  |

**Table S5.** Double-stranded DNA fragments (gBlocks) used as standard curve for qPCR analysis.

| Assay | Sequence |
| --- | --- |
| *bla*_CTX-M_ | GGTACCGAGAACCTGTACTTCCAATCCAATATGGTTAAAAAATCACTGCGTCAGTTCACGCTGATGGCGACGGCAACCGTCACGCTGTTGTTAGGAAGTGTGCCGCTGTATGCGCAAACGGCGGACGTACAGCAAAAACTTGCCGAATTAGAGCGGCAGTCGGGAGGAAGACTGGGTGTGGCATTGATTAACACAGCAGATAATTCGCAAATACTTTATCGTGCTGATGAGCGCTTTGCGATGTGCAGCACCAGTAAAGTGATGGCCGTGGCCGCGGTGCTGAAGAAAAGTGAAAGCGAACCGAATCTGTTAAATCAGCGAGTTGAGATCAAAAAATCTGACTTGGTTAACTATAATCCGATTGCGGAAAAGCACGTCGATGGGACGATGTCACTGGCTGAGCTTAGCGCGGCCGCGCTACAGTACAGCGATAACGTGGCGATGAATAAGCTGATTTCTCACGTTGGCGGCCCGGCTAGCGTCACCGCGTTCGCCCGACAGCTGGGAGACGAAACGTTCCGTCTCGACCGTACCGAGCCGACGTTAAACACCGCCATTCCGGGCGATCCGCGTGATACCACTTCACCTCGGGCAATGGCGCAAACTCTGCGTAATCTGACGCTGGGTAAAGCATTGGGTGACAGCCAACGGGCGCAGCTGGTGACATGGATGAAAGGCAATACCACCGGTGCAGCGAGCATTCAGGCTGGACTGCCTGCTTCCTGGGTTGTGGGGGATAAAACCGGCAGCGGTGACTATGGCACCACCAACGATATCGCGGTGATCTGGCCAAAAGATCGTGCGCCGCTGATTCTGGTCACTTACTTCACCCAGCCTCAACCTAAGGCAGAAAGCCGTCGCGATGTATTAGCGTCGGCGGCTAAAATCGTCACCAACGGTTTGTAAATTGGAAGTGGATAACGGATCCGAATTCGA |
| *bla*_KPC_ | GGTACCGAGAACCTGTACTTCCAATCCAATATGTCACTGTATCGCCGTCTAGTTCTGCTGTCTTGTCTCTCATGGCCGCTGGCTGGCTTTTCTGCCACCGCGCTGACCAACCTCGTCGCGGAACCATTCGCTAAACTCGAACAGGACTTTGGCGGCTCCATCGGTGTGTACGCGATGGATACCGGCTCAGGCGCAACTGTAAGTTACCGCGCTGAGGAGCGCTTCCCACTGTGCAGCTCATTCAAGGGCTTTCTTGCTGCCGCTGTGCTGGCTCGCAGCCAGCAGCAGGCCGGCTTGCTGGACACACCCATCCGTTACGGCAAAAATGCGCTGGTTCCGTGGTCACCCATCTCGGAAAAATATCTGACAACAGGCATGACGGTGGCGGAGCTGTCCGCGGCCGCCGTGCAATACAGTGATAACGCCGCCGCCAATTTGTTGCTGAAGGAGTTGGGCGGCCCGGCCGGGCTGACGGCCTTCATGCGCTCTATCGGCGATACCACGTTCCGTCTGGACCGCTGGGAGCTGGAGCTGAACTCCGCCATCCCAGGCGATGCGCGCGATACCTCATCGCCGCGCGCCGTGACGGAAAGCTTACAAAAACTGACACTGGGCTCTGCACTGGCTGCGCCGCAGCGGCAGCAGTTTGTTGATTGGCTAAAGGGAAACACGACCGGCAACCACCGCATCCGCGCGGCGGTGCCGGCAGACTGGGCAGTCGGAGACAAAACCGGAACCTGCGGAGTGTATGGCACGGCAAATGACTATGCCGTCGTCTGGCCCACTGGGCGCGCACCTATTGTGTTGGCCGTCTACACCCGGGCGCCTAACAAGGATGACAAGTACAGCGAGGCCGTCATCGCCGCTGCGGCTAGACTCGCGCTCGAGGGATTGGGCGTCAACGGGCAGTAAGGCTCTGAAAATCATCTATTGGCCCACCACCGCCGCCCTTGCGGGCGGCATGGATTACCAACCACTGTCACATTTAGGATTGGAAGTGGATAACGGATCCGAATTCGA |
| *bla*_NDM_ | GGTACCGAGAACCTGTACTTCCAATCCAATATGGAATTGCCCAATATTATGCACCCGGTCGCGAAGCTGAGCACCGCATTAGCCGCTGCATTGATGCTGAGCGGGTGCATGCCCGGTGAAATCCGCCCGACGATTGGCCAGCAAATGGAAACTGGCGACCAACGGTTTGGCGATCTGGTTTTCCGCCAGCTCGCACCGAATGTCTGGCAGCACACTTCCTATCTCGACATGCCGGGTTTCGGGGCAGTCGCTTCCAACGGTTTGATCGTCAGGGATGGCGGCCGCGTGCTGGTGGTCGATACCGCCTGGACCAATGACCAGACCGCCCAGATCCTCAACTGGATCAAGCAGGAGATCAACCTGCCGGTCGCGCTGGCGGTGGTGACTCACGCGCATCAGGACAAGATGGGCGGTATGGACGCGCTGCATGCGGCGGGGATTGCGACTTATGCCAATGCGTTGTCGAACCAGCTTGCCCCGCAAGAGGGGATGGTTGCGGCGCAACACAGCCTGACTTTCGCCGCCAATGGCTGGGTCGAACCAGCAACCGCGCCCAACTTTGGCCCGCTCAAGGTATTTTACCCCGGCCCCGGCCACACCAGTGACAATATCACCGTTGGGATCGACGGCACCGACATCGCTTTTGGTGGCTGCCTGATCAAGGACAGCAAGGCCAAGTCGCTCGGCAATCTCGGTGATGCCGACACTGAGCACTACGCCGCGTCAGTGCGCGCGTTTGGTGCGGCGTTCCCCAAGGCCAGCATGATCGTGATGAGCCATTCCGCCCCCGATAGCCGCGCCGCAATCACTCATACGGCCCGCATGGCCGACAAGCTGCGCTGAATTGGAAGTGGATAACGGATCCGAATTCGA |
| *vanA* | GGTACCGAGAACCTGTACTTCCAATCCAATATGAATAGAATAAAAGTTGCAATACTGTTTGGGGGTTGCTCAGAGGAGCATGACGTATCGGTAAAATCTGCAATAGAGATAGCCGCTAACATTAATAAAGAAAAATACGAGCCGTTATACATTGGAATTACGAAATCTGGTGTATGGAAAATGTGCGAAAAACCTTGCGCGGAATGGGAAAACGACAATTGCTATTCAGCTGTACTCTCGCCGGATAAAAAAATGCACGGATTACTTGTTAAAAAGAACCATGAATATGAAATCAACCATGTTGATGTAGCATTTTCAGCTTTGCATGGCAAGTCAGGTGAAGATGGATCCATACAAGGTCTGTTTGAATTGTCCGGTATCCCTTTTGTAGGCTGCGATATTCAAAGCTCAGCAATTTGTATGGACAAATCGTTGACATACATCGTTGCGAAAAATGCTGGGATAGCTACTCCCGCCTTTTGGGTTATTAATAAAGATGATAGGCCGGTGGCAGCTACGTTTACCTATCCTGTTTTTGTTAAGCCGGCGCGTTCAGGCTCATCCTTCGGTGTGAAAAAAGTCAATAGCGCGGACGAATTGGACTACGCAATTGAATCGGCAAGACAATATGACAGCAAAATCTTAATTGAGCAGGCTGTTTCGGGCTGTGAGGTCGGTTGTGCGGTATTGGGAAACAGTGCCGCGTTAGTTGTTGGCGAGGTGGACCAAATCAGGCTGCAGTACGGAATCTTTCGTATTCATCAGGAAGTCGAGCCGGAAAAAGGCTCTGAAAACGCAGTTATAACCGTTCCCGCAGACCTTTCAGCAGAGGAGCGAGGACGGATACAGGAAACGGCAAAAAAAATATATAAAGCGCTCGGCTGTAGAGGTCTAGCCCGTGTGGATATGTTTTTACAAGATAACGGCCGCATTGTACTGAACGAAGTCAATACTCTGCCCGGTTTCACGTCATACAGTCGTTATCCCCGTATGATGGCCGCTGCAGGTATTGCACTTCCCGAACTGATTGACCGCTTGATCGTATTAGCGTTAAAGGGGTGAATTGGAAGTGGATAACGGATCCGAATTCGA |
| *C. difficile* 16S rRNA gene | GGTACCGAGAACCTGTACTTCCAATCCAATGAGTTTGATCCTGGCTCAGGATGAACGCTGGCGGCGTGCCTAACACATGCAAGTTGAGCGATTTACTTCGGTAAAGAGCGGCGGACGGGTGAGTAACGCGTGGGTAACCTACCCTGTACACACGGATAACATACCGAAAGGTATGCTAATACGGGATAATATATTTGAGAGGCATCTCTTGAATATCAAAGGTGAGCCAGTACAGGATGGACCCGCGTCTGATTAGCTAGTTGGTAAGGTAACGGCTTACCAAATCACTATCAAAATAAAAGTGTTTACCATCAATAGTTTTATAACCATTAAAGGCAATAGCGGTATCAGTATCAAAGTAGTATCTACTACCATTAACAGTCTGCCAACCTTTTGAGATGATAGCAGTGTCAGGATTGAAATAATACTTATTATTGTCAATTACTTGCAATCCGACTGCAGCAATAGCATTATTAGGATTAAAGTAATATTTCATTGGAAGTGGATAACGGATCCGAATTCGA |
| *C. difficile* *tcdA* |  |

**SUPPLEMENTARY FIGURES**

**
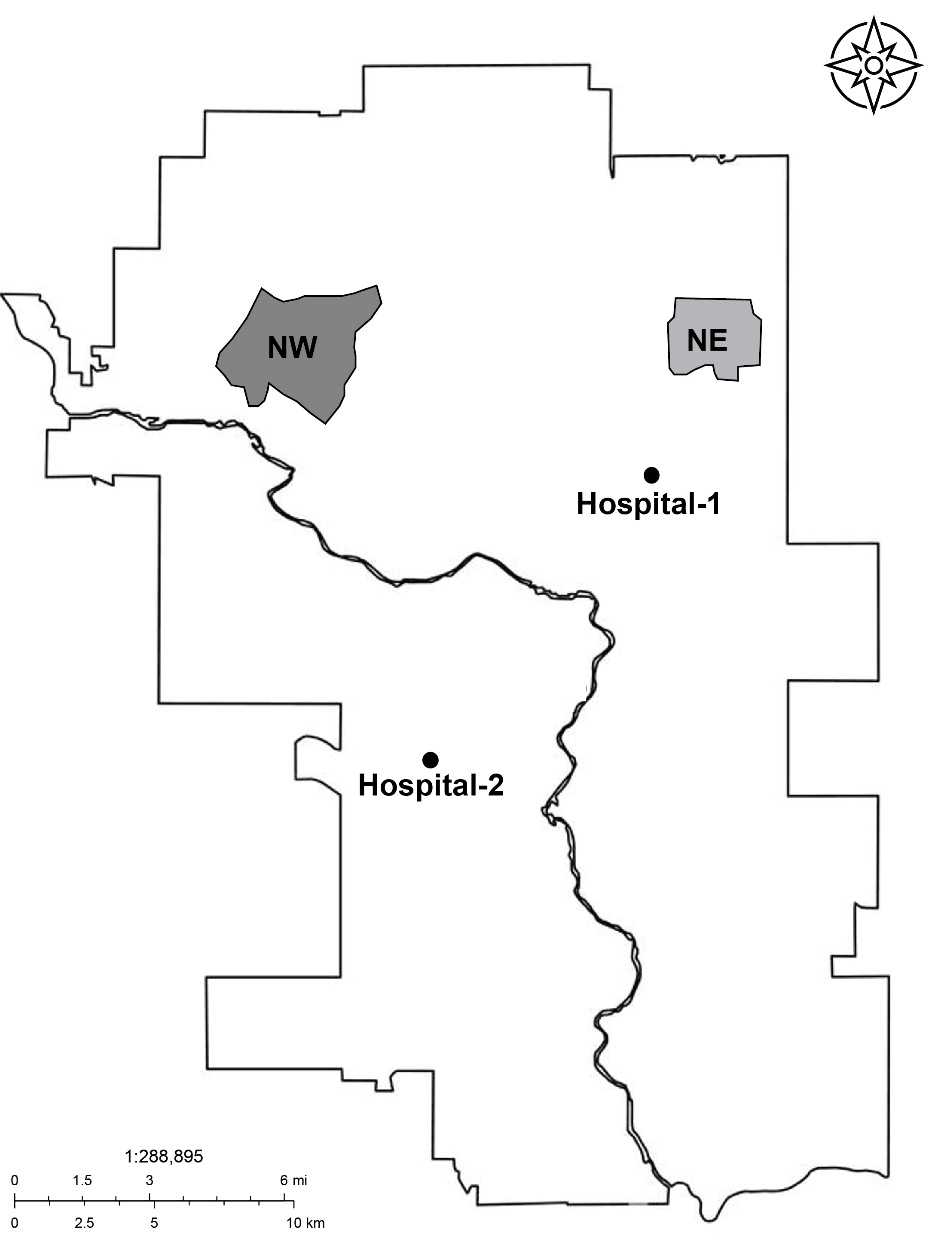
**

**Figure S1. Map of Calgary showing the two hospitals and the two targeted neighborhoods included in the study.** Hospital locations are indicted as dots and neighborhoods’ catchment area are shown by the smaller shaded regions and are designated by quadrant: NE and NW.

**
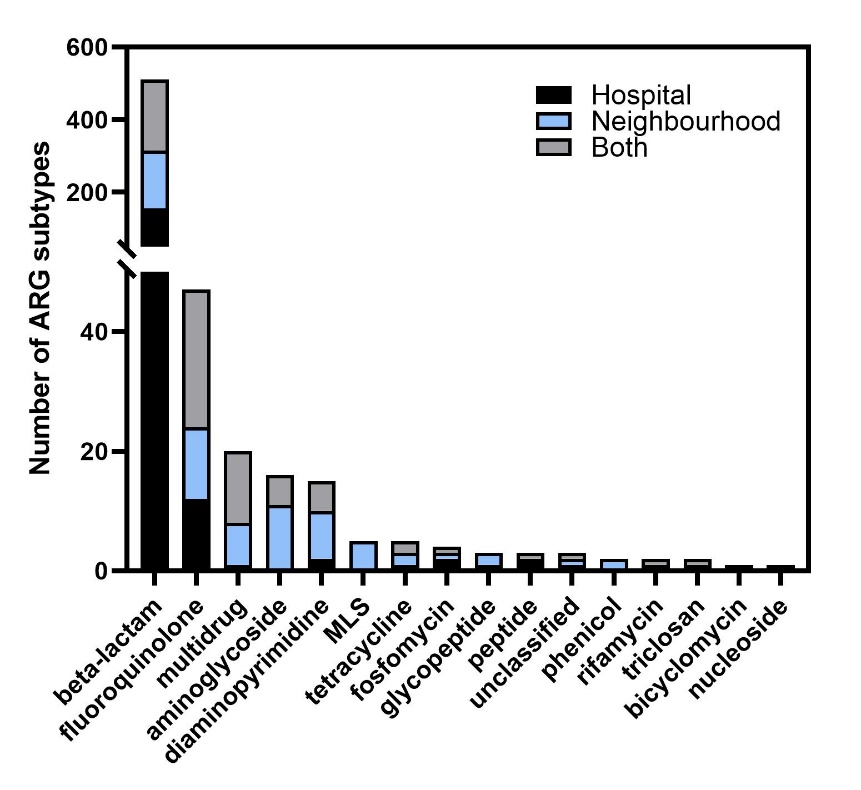
**

**Figure S2.** **Cumulative ARGs only detected after culture-enrichment for at least one of the sites.** Percentage of ARG subtypes that were detected through culture-enrichment in one or more samples and undetected in corresponding raw wastewater. ARGs were classified by target antibiotic and categorized based on location type from which samples were collected (i.e., Hospital and neighborhood).

**
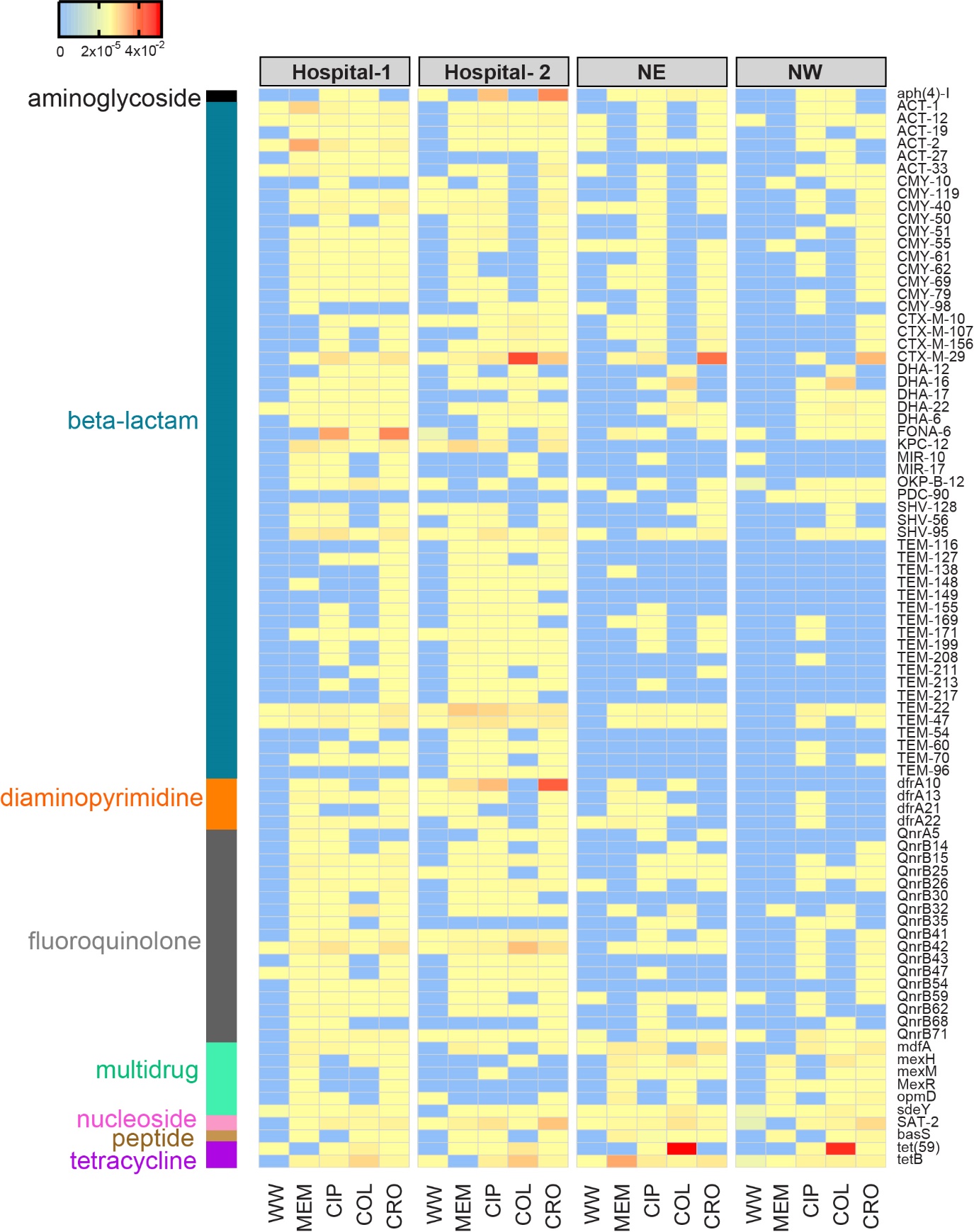
**

**Figure S3. Heatmap of metagenomic profiles of cultured-enriched ARGs that were commonly identified after antibiotic impregnated semi-selective culture enrichment.** Relative abundances (ARGs per 16S rRNA gene) of 86 ARGs that were only detected after culture-enrichment in one or more samples and were common across all antibiotic conditions. ARGs are organized and color coded by target antibiotic (left bar). WW: raw wastewater, MEM: meropenem, CIP: ciprofloxacin, COL: colistin and CRO: ceftriaxone.


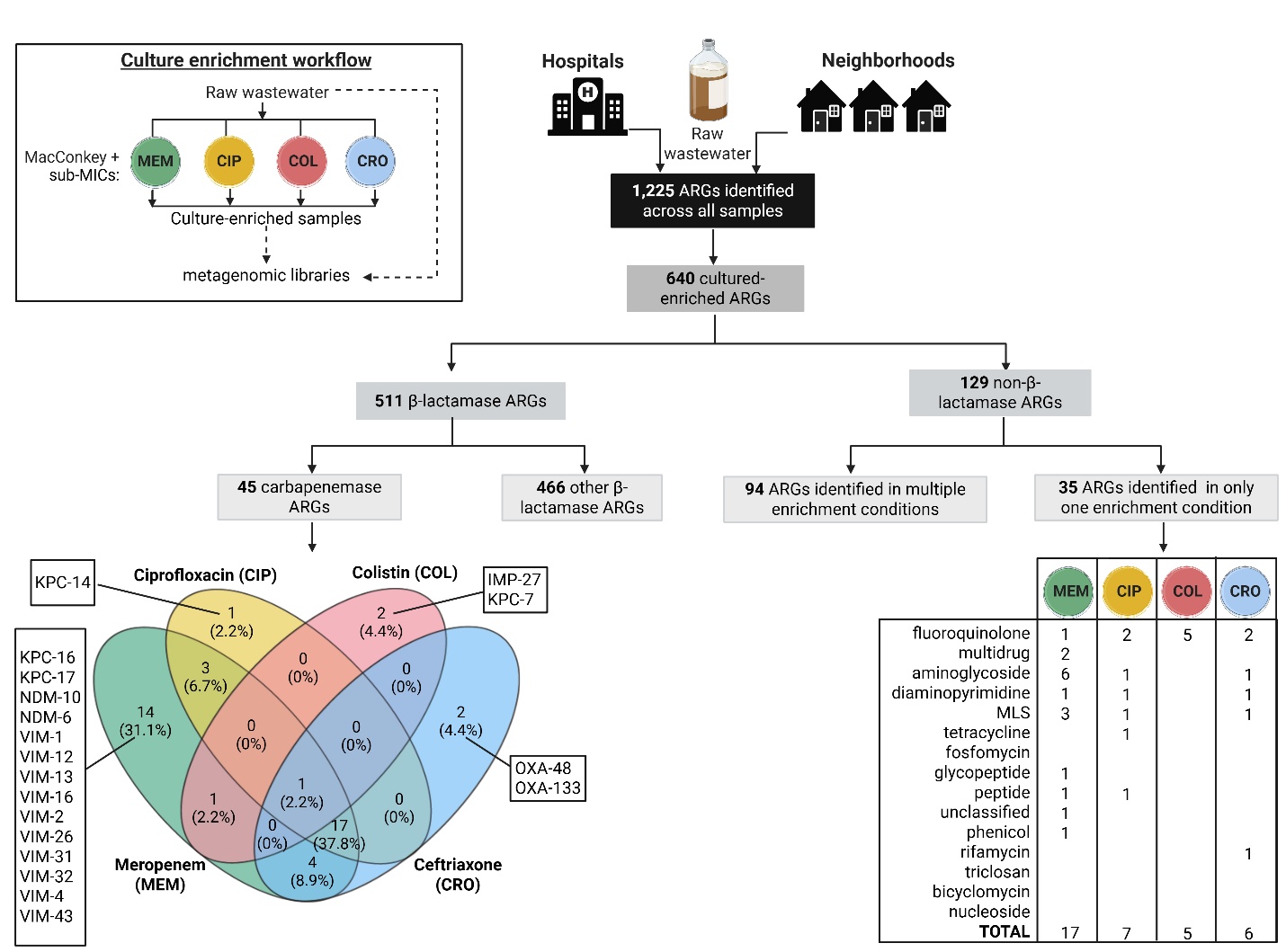


**Figure S4.**  **Workflow for ARG detection profile in wastewater focusing on carbapenemases and enrichment condition efficiency.** Overview of the number and types of ARGs identified from wastewater samples cultured under four different antibiotic enrichment conditions. Among the 1225 identified ARG subtypes, 640 were only detected through culture-enrichment at one or more sites, where the ARGs was undetected in the raw wastewater sample and only detected after at least one enrichment condition (termed as cultured-enriched ARGs). Among these, 511 were β-lactamase genes, including 45 carbapenemases presented in the Venn diagram (individual ARGs unique to each antibiotic enrichment condition are stated in each box; relative abundances of the carbapenemases are detailed in Fig. S9). The remaining 129 non-β-lactamase ARGs were further classified based on whether they were exclusive to a single enrichment condition or shared across multiple. Enrichment with meropenem resulted in the greatest identification and diversity of carbapenemases and non-beta-lactamase ARGs.


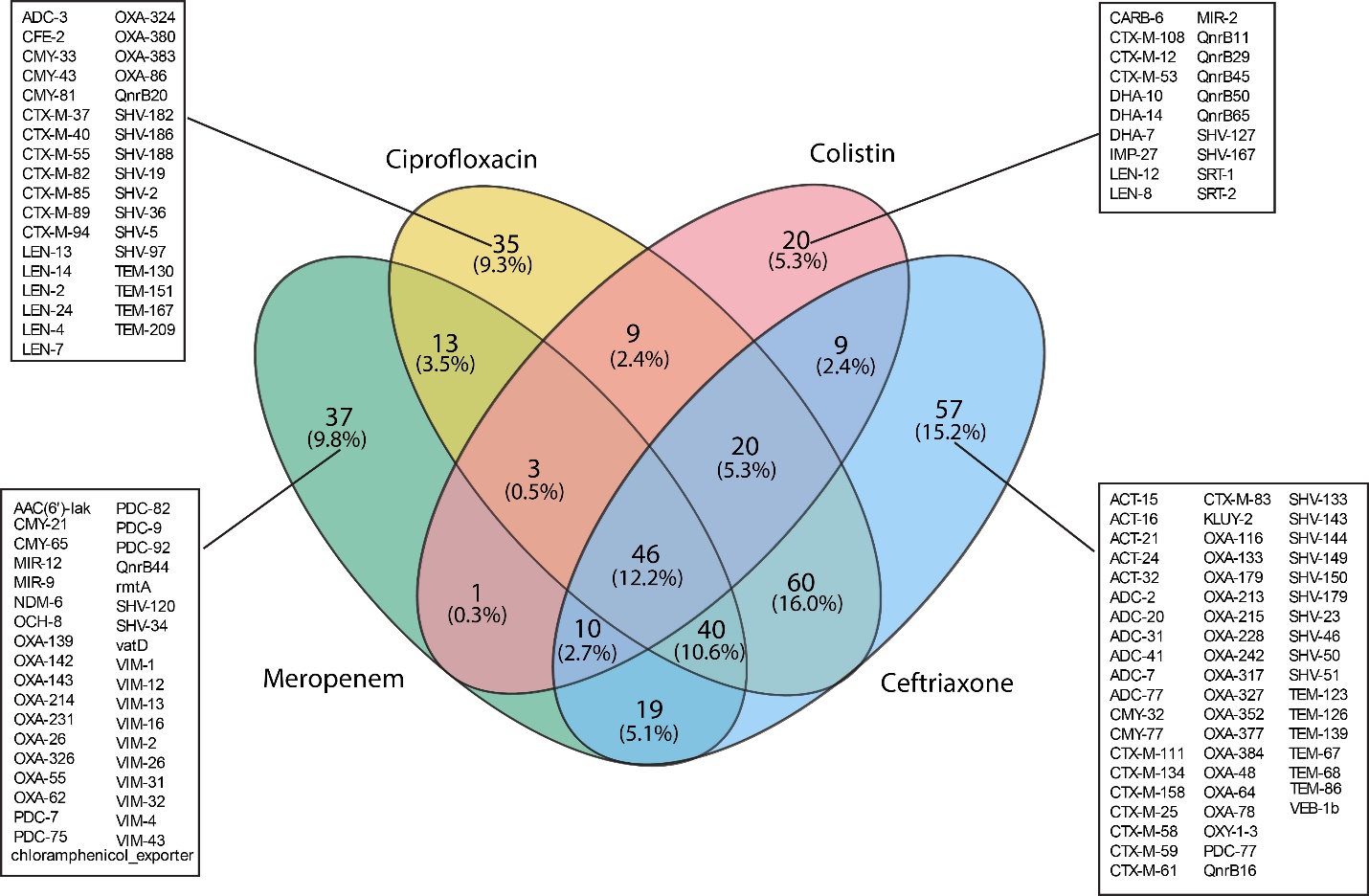


**Figure S5.**  **ARGs exclusively found through culture enrichment classified by the antibiotic used for selection.** Venn diagram showing 376 ARG subtypes that were not identified in raw samples across all sites and were only detected through culture enrichment. ARGs are classified by the antibiotic used for culture enrichment. Individual ARGs unique to each antibiotic enrichment condition are stated in each box.

**
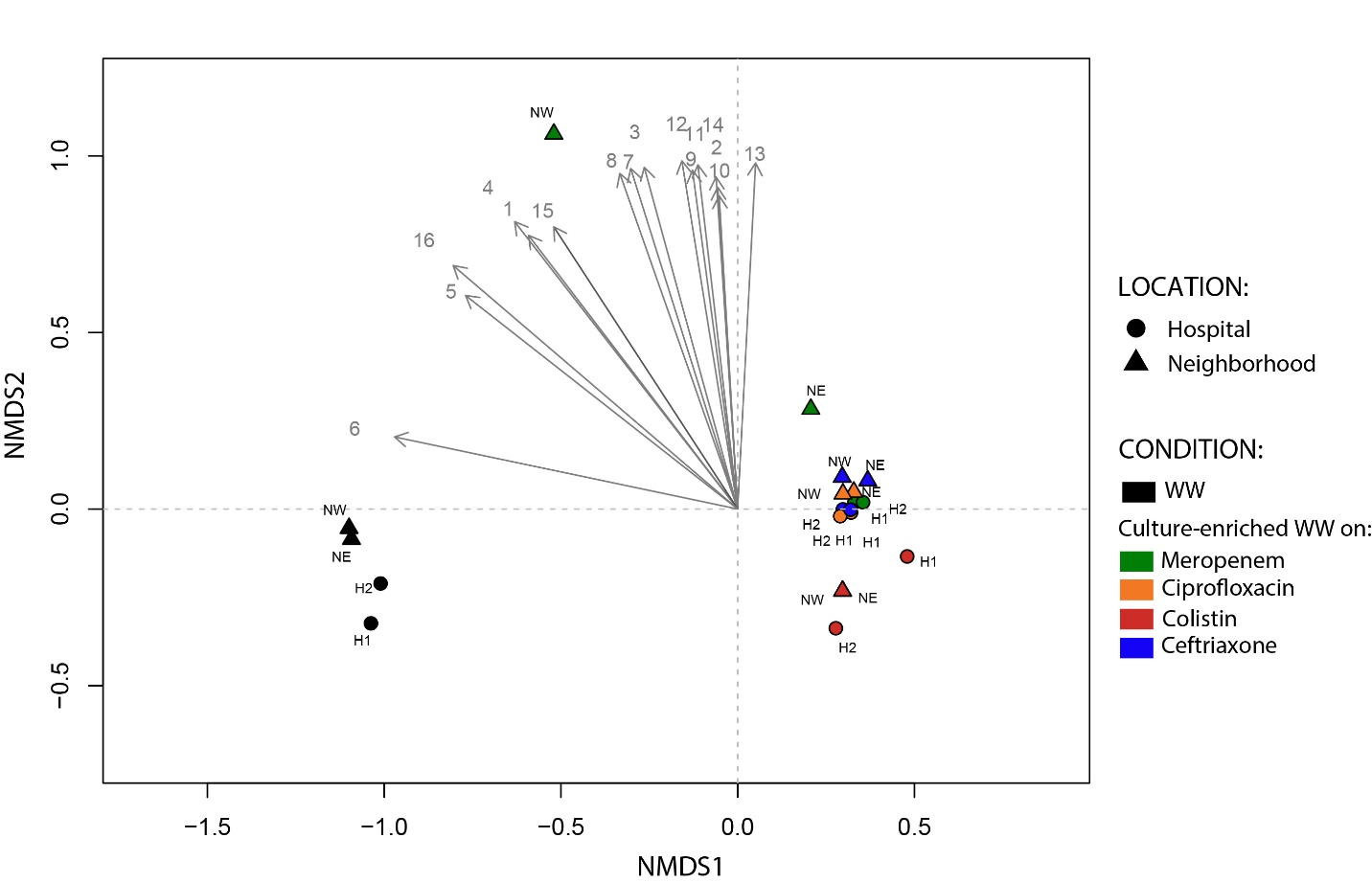
**

**Figure S6. The ARG subtypes significantly correlated with the ordination for wastewater resistome before and after culture enrichment for sample NW-Meropenem (Figure 3) (P≤0.001).** The ARG subtypes that were strongly associated with NW-meropenem (i.e., associated with 1^st^ and 2^nd^ quadrants) are colored in grey and displayed in vectors. 1: *ant*(9)-I, 2: *aph*(3')-IIb, 3: *efrA*, 4: *efrB*, 5: *erm*(TR), 6: *lmrD*, 7: *lsa*, 8: *optrA*, 9: *smeB*, 10: *smeC*, 11: *smeD*, 12: *smeE*, 13: *smeF*, 14: *smeS*, 15: *tetL*, and 16: *arlR*. H1: Hospital-1, H2: Hospital-2.


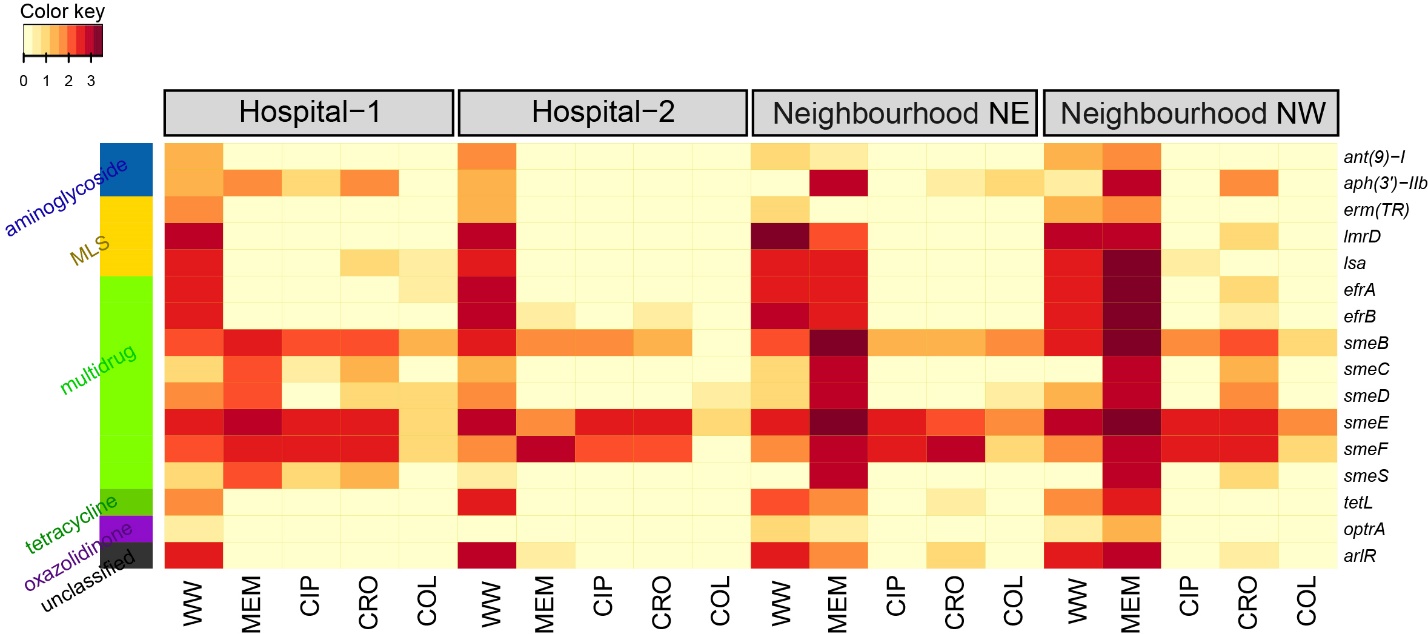


**Figure S7. Heatmap for log-transformed relative abundances of key ARG subtypes that were significantly correlated with the ordination in Figure 5S and associated with NW-MEM.** Unit: Log_10_(concentration × 10^5^ + 1). ARGs are classified by target antibiotic (left bar). WW: wastewater, MEM: meropenem, CIP: ciprofloxacin, CRO: ceftriaxone and COL: colistin.


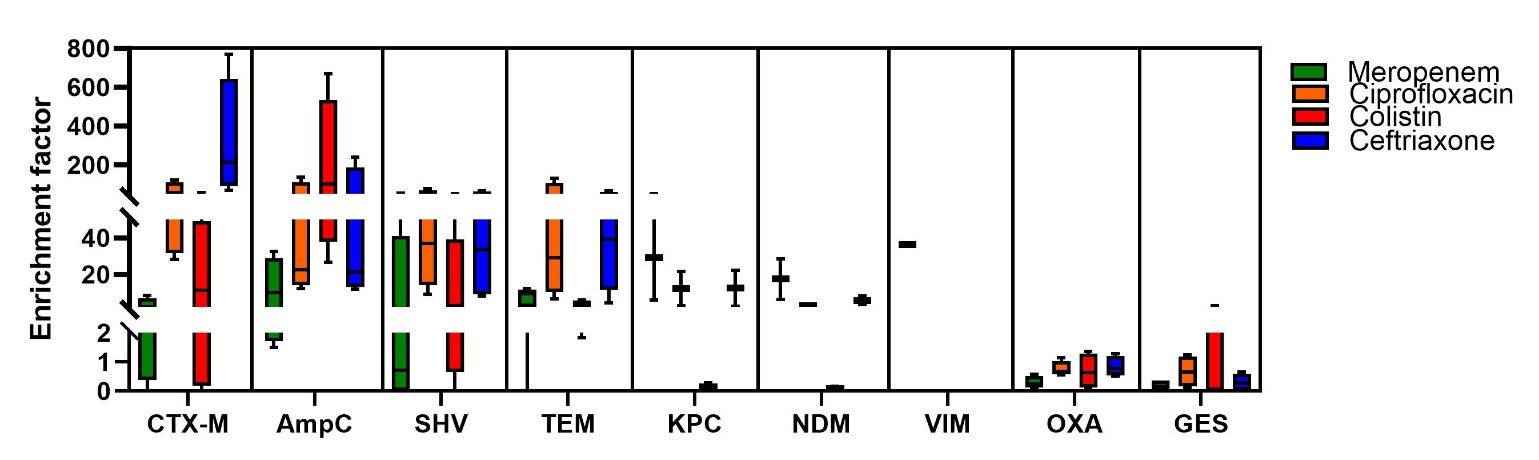


**Figure S8. Enrichment factor ratio for β-lactamases ARGs as a function of semi-selective culture condition.** Enrichment factor (EF) was calculated as the metagenomic abundance ratio of a given β-lactamase:16S rRNA genes following growth on MacConkey agar plates impregnated with antibiotics (meropenem, ciprofloxacin, ceftriaxone and colistin) relative to raw wastewater in each sample. Median and interquartile ranges are indicated as the middle, top, and bottom lines of each box. Ends of the whiskers mark the 10 and 90 percentile EF determined in each category.

**
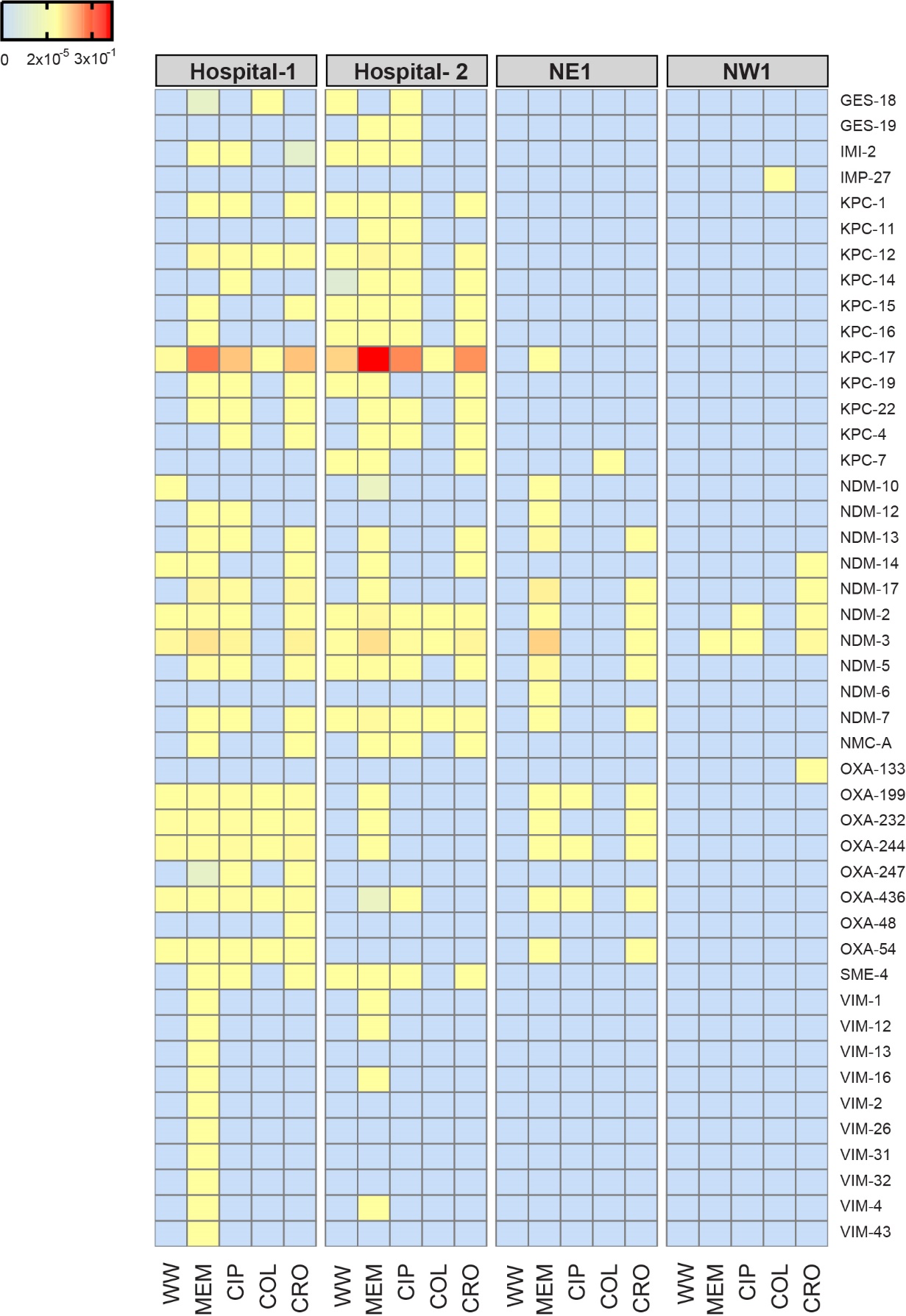
**

**Figure S9. Heatmap of carbapenemase ARGs enriched after semi-selective culture enrichment relative to raw wastewater.** Relative abundances (genes per 16S rRNA gene) of carbapenemases that were only detected after culture-enrichment in one or more samples. WW: raw wastewater, MEM: meropenem, CIP: ciprofloxacin, COL: colistin and CRO: ceftriaxone.


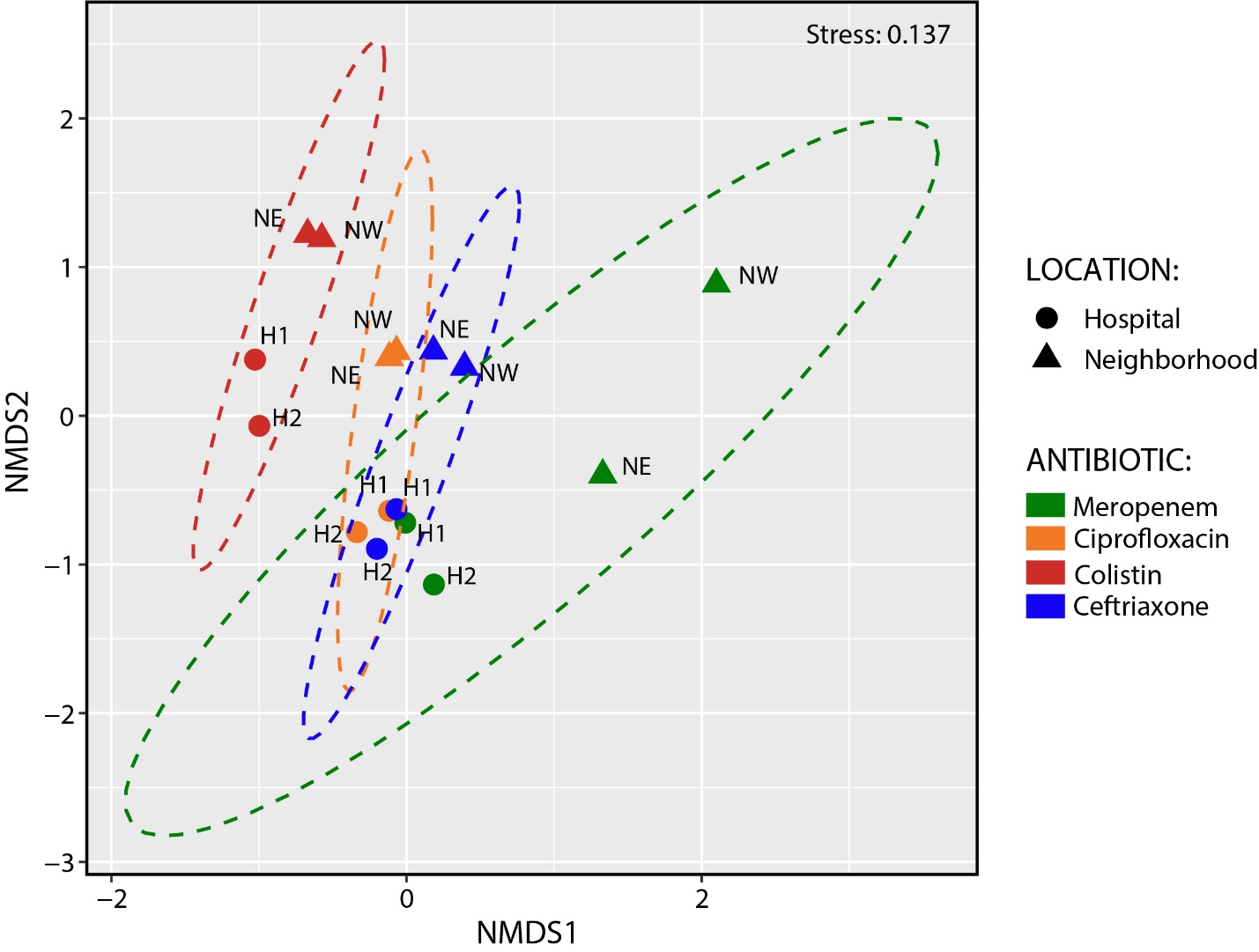


**Figure S10. Beta diversity of cultured-enriched ARGs from wastewater resistome.** NMDS plot based on the Bray-Curtis distances of cultured-enriched ARGs profiles. Ellipses denote 95% confidence intervals for the multivariate distribution. Samples were shape-coded based on the type of location where the sample was collected [i.e., hospital (H1: Hospital-1 and H2: Hospital-2) or neighborhood (NE and NW)]; and color-coded based on the antibiotic used for selection.

**
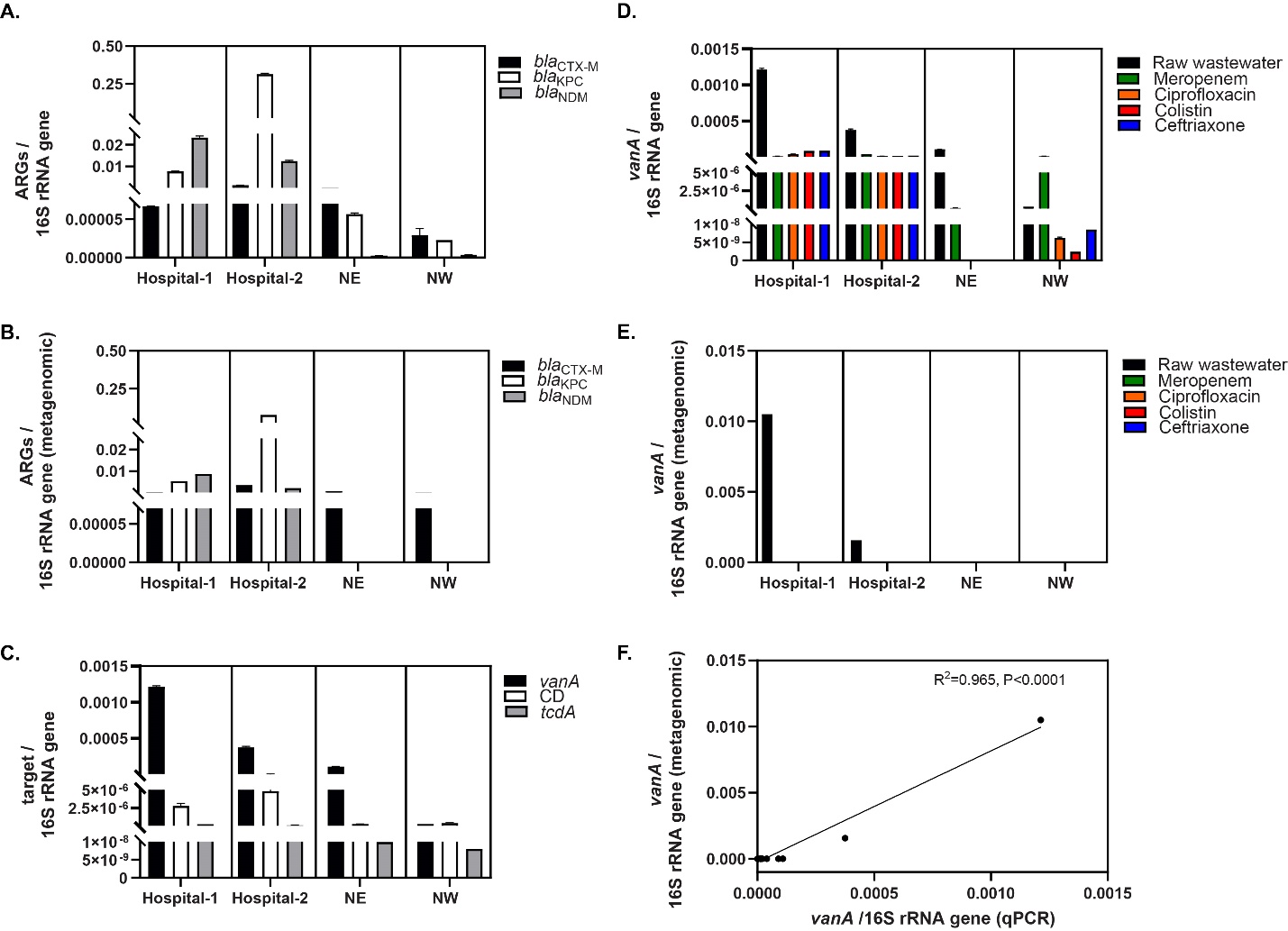
**

**Figure S11.** **Correlating ARGs identified in raw wastewater by metagenomics with independent qPCR.** **A.** Absolute quantification of targeted beta-lactamases normalized by the total bacterial load in raw wastewater collected from hospitals (Hospital-1 and Hospital-2) and neighborhoods (NE and NW). **B.** Normalized ARG abundance from metagenomic is expressed as numbers of ARG per 16S rRNA gene of targeted beta-lactamases in raw wastewater collected from hospitals and neighborhoods. **C.** Absolute quantification of nosocomial Gram positive pathogens and ARO; *vanA* (for total burden of vancomycin-resistant *Enterococcus* (VRE)) and *Clostridioides difficile* (CD) by targeted qPCR normalized by the total bacterial load in raw wastewater samples. **D.** Absolute quantification of *vanA* by qPCR normalized by the total bacterial load in raw wastewater and culture-enriched samples. **E.** Normalized abundance from metagenomic data of *vanA* in raw wastewater and culture-enriched samples. **F.** An example of the correlation between qPCR and metagenome data illustrated using *vanA*. Best-fit line was generated using a simple linear model.

**REFERENCES**

1. Arango-Argoty, G.*, et al.* DeepARG: a deep learning approach for predicting antibiotic resistance genes from metagenomic data. *Microbiome* **6**, 23 (2018).

2. Yang, Y.*, et al.* ARGs-OAP: online analysis pipeline for antibiotic resistance genes detection from metagenomic data using an integrated structured ARG-database. *Bioinformatics* **32**, 2346-2351 (2016).

3. Marathe, N.P.*, et al.* Sewage effluent from an Indian hospital harbors novel carbapenemases and integron-borne antibiotic resistance genes. *Microbiome* **7**, 97 (2019).

4. Keenum, I.*, et al.* Combined effects of composting and antibiotic administration on cattle manure–borne antibiotic resistance genes. *Microbiome* **9**, 81 (2021).

5. Prieto Riquelme, M.V.*, et al.* Demonstrating a Comprehensive Wastewater-Based Surveillance Approach That Differentiates Globally Sourced Resistomes. *Environmental Science & Technology* **56**, 14982-14993 (2022).

6. Zhao, J.*, et al.* Decade-long bacterial community dynamics in cystic fibrosis airways. *Proc Natl Acad Sci U S A* **109**, 5809-5814 (2012).

7. EUCAST. 2025. The European Committee on Antimicrobial Susceptibility Testing: Clinical breakpoints - breakpoints and guidance. https://www.eucast.org/fileadmin/src/media/PDFs/EUCAST_files/Breakpoint_tables/v_15.0_Breakpoint_Tables.pdf. Accessed March 1 2025.

8. Lewis II JS. 2025. Clinical and Laboratory Standards Institute: Performance standards for antimicrobial susceptibility testing. CLSI document M100.

9. Alberta-Precision-Laboratories. 2022. Antibiograms: Calgary zone, on https://www.albertaprecisionlabs.ca/hp/Page13779.aspx. Accessed April 3 2025.

10. Kubota, H.*, et al.* Development of TaqMan-Based Quantitative PCR for Sensitive and Selective Detection of Toxigenic Clostridium difficile in Human Stools. *PLOS ONE* **9**, e111684 (2014).

11. Nadkarni, M.A., Martin, F.E., Jacques, N.A. & Hunter, N. Determination of bacterial load by real-time PCR using a broad-range (universal) probe and primers set. *Microbiology* **148**, 257-266 (2002).
